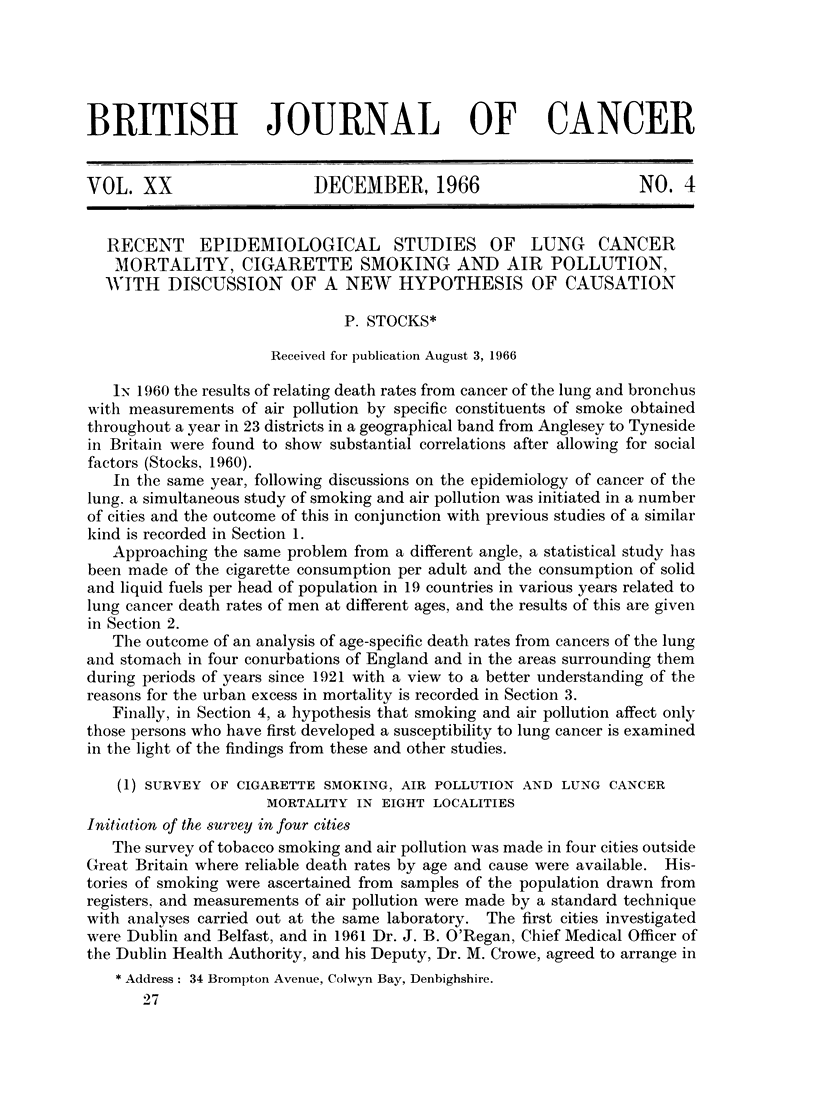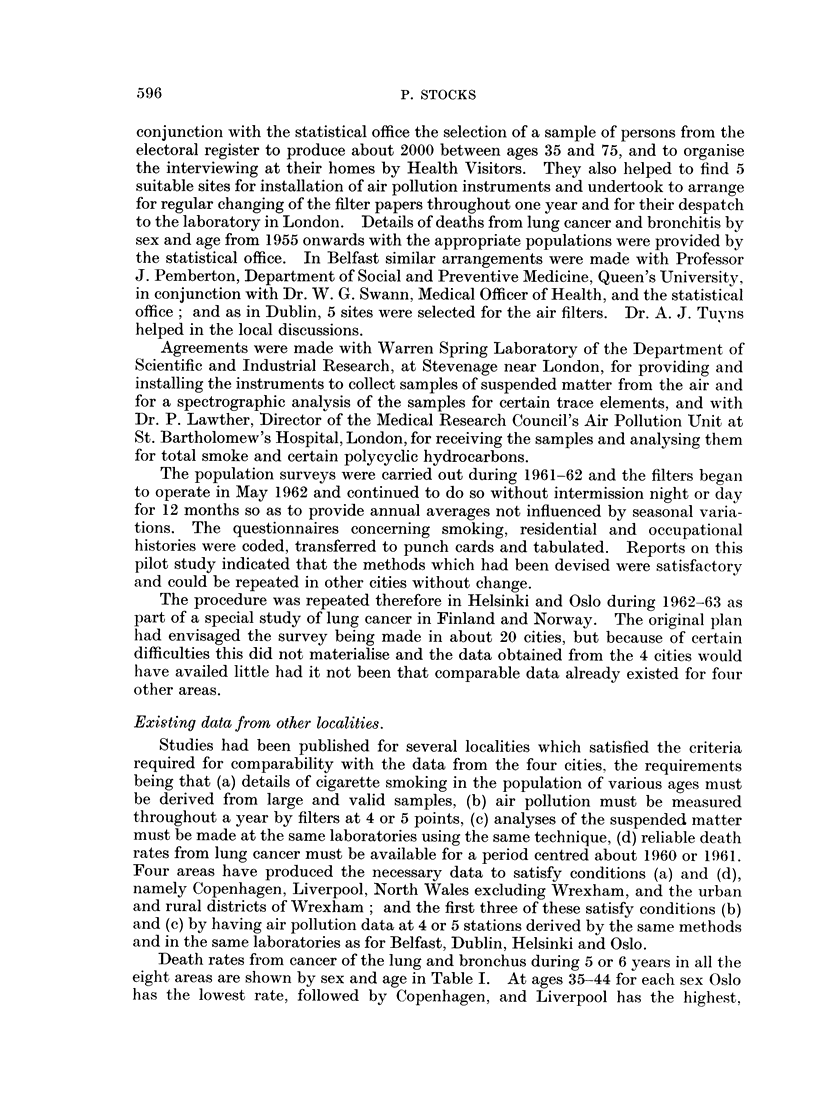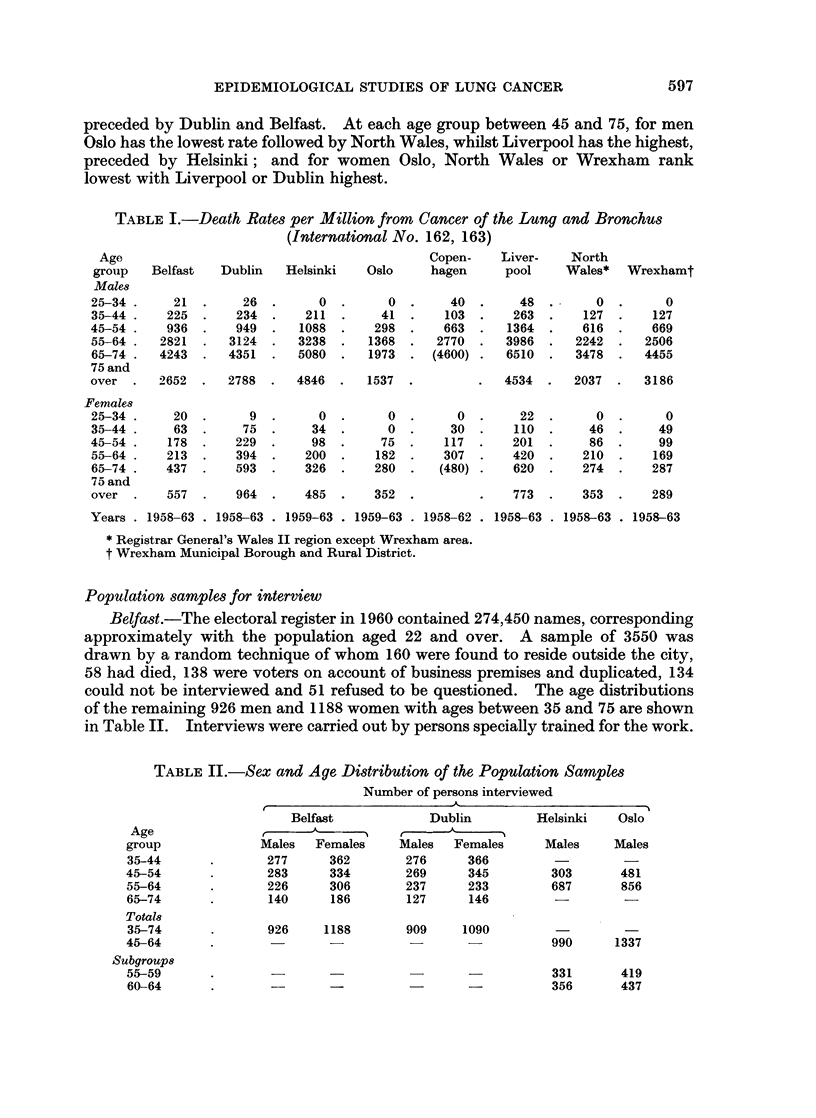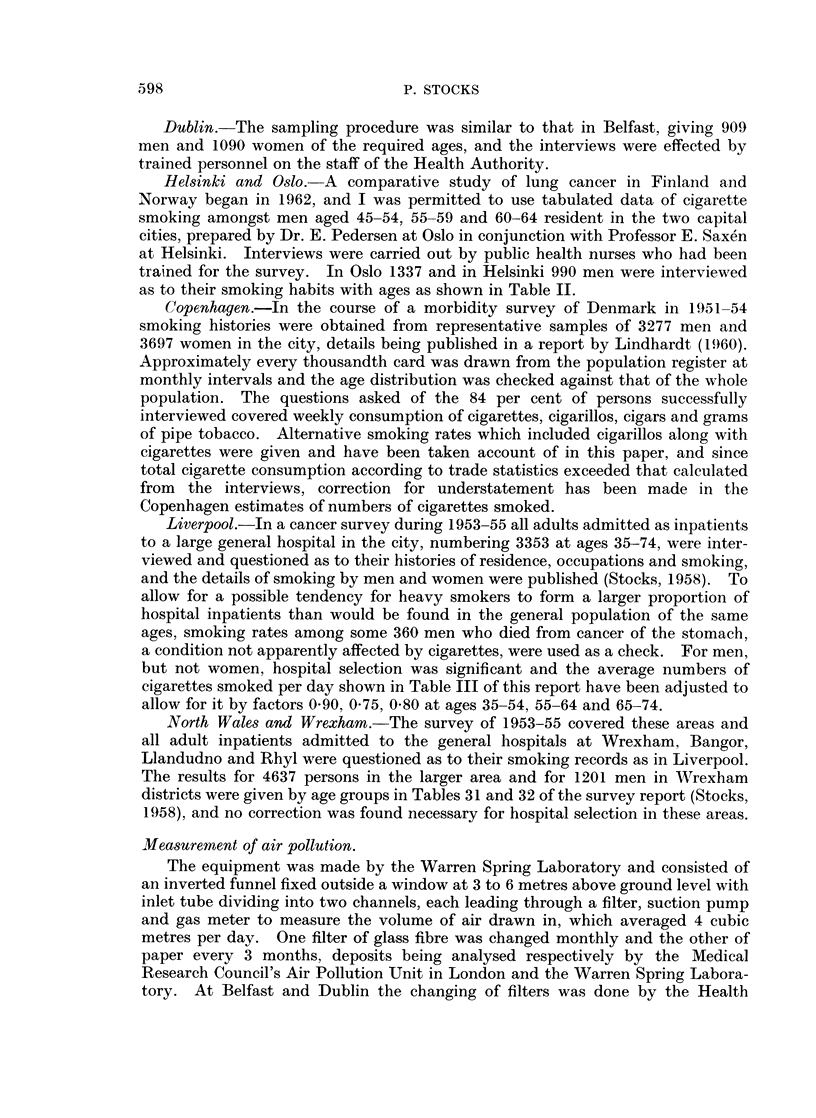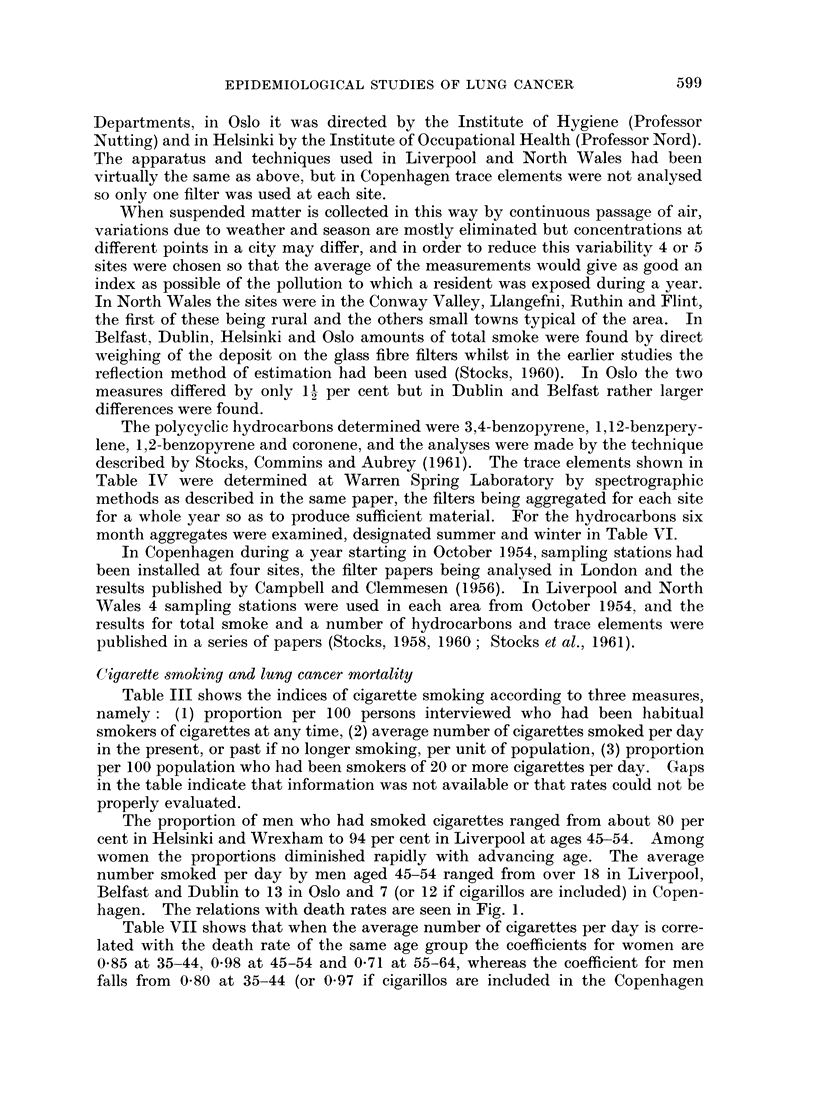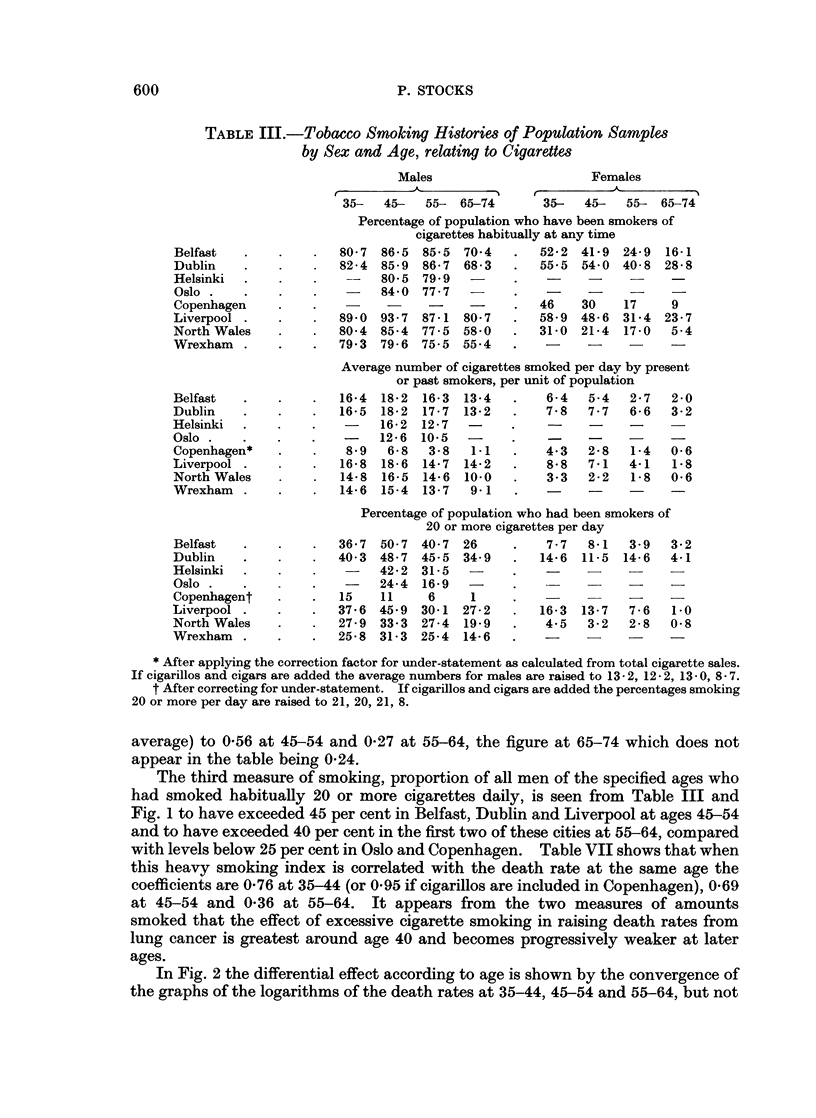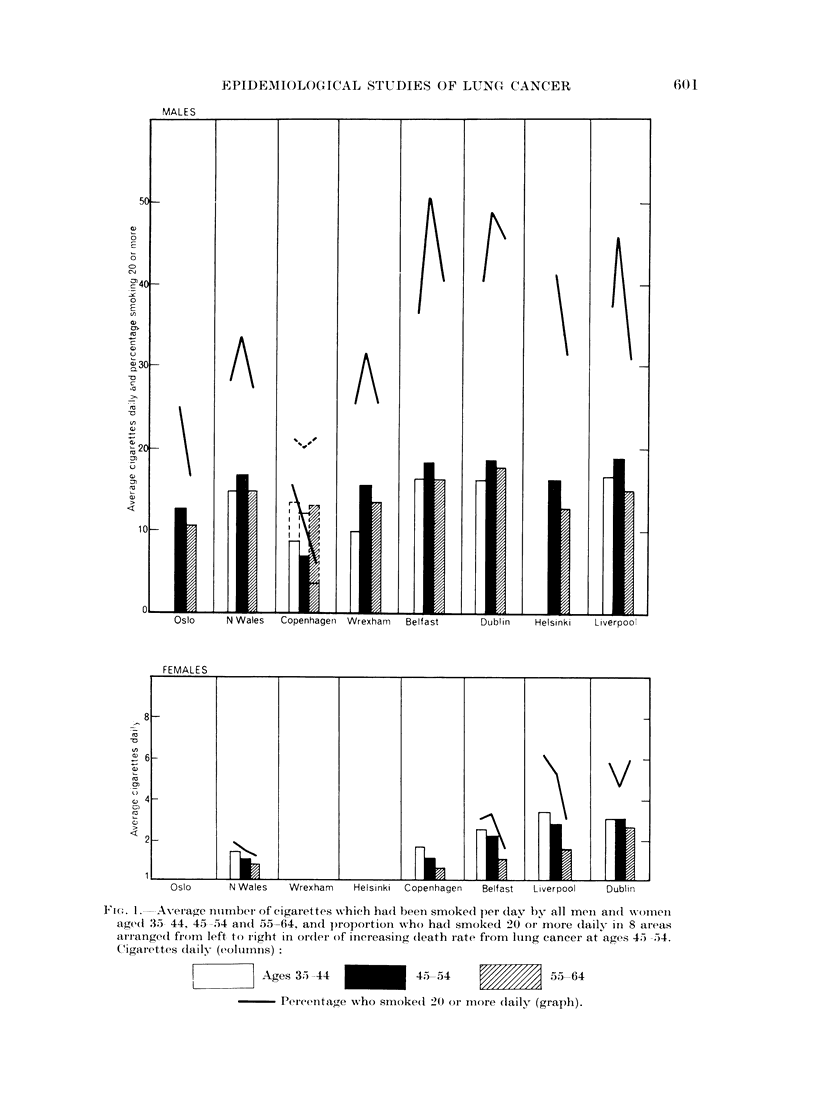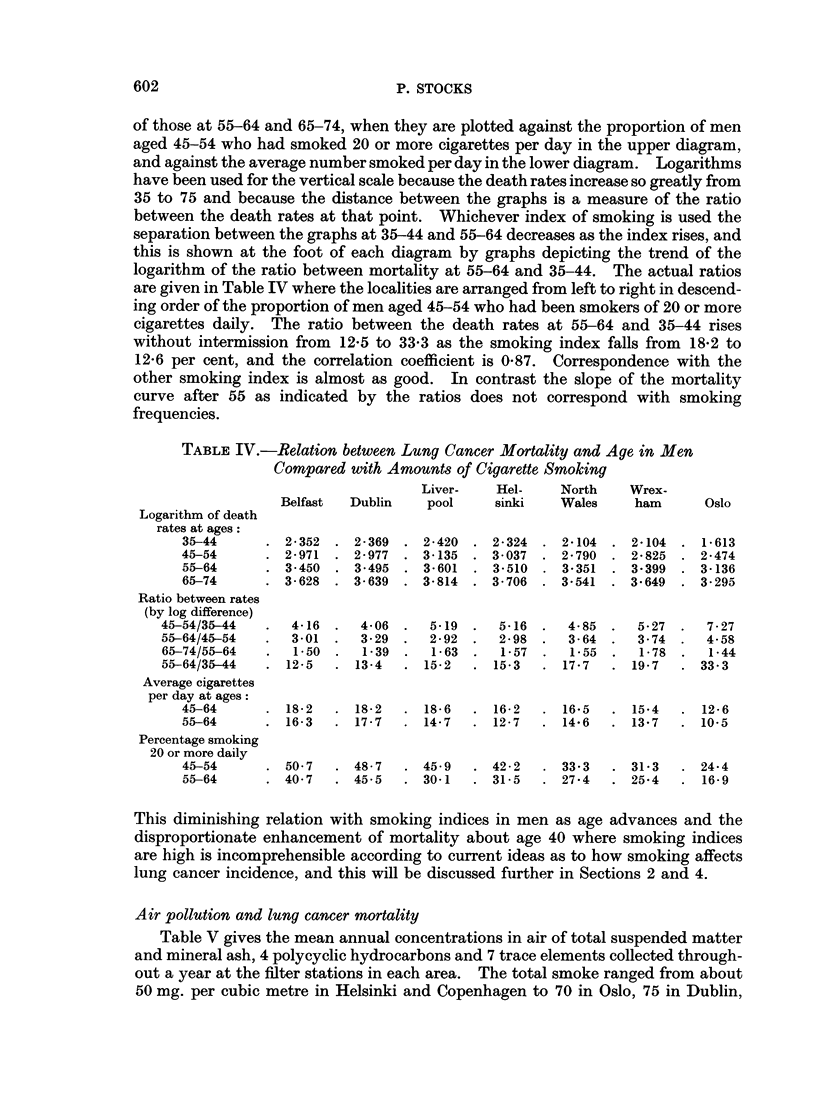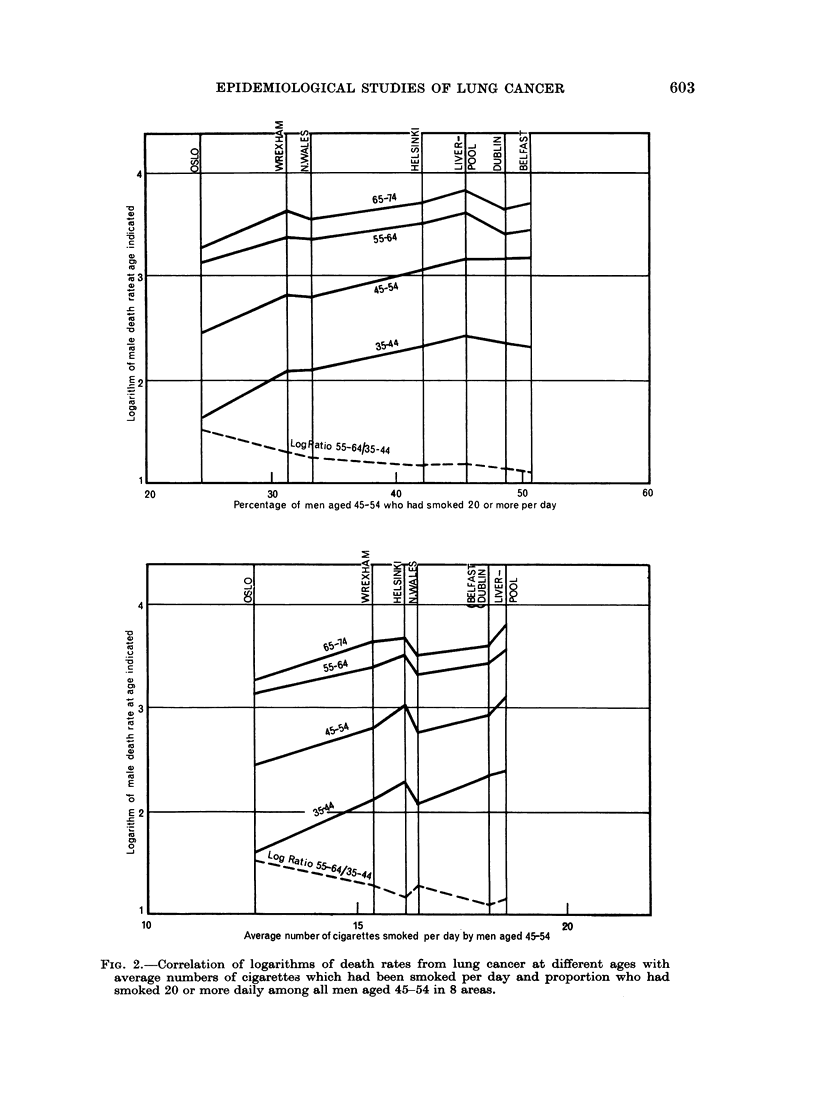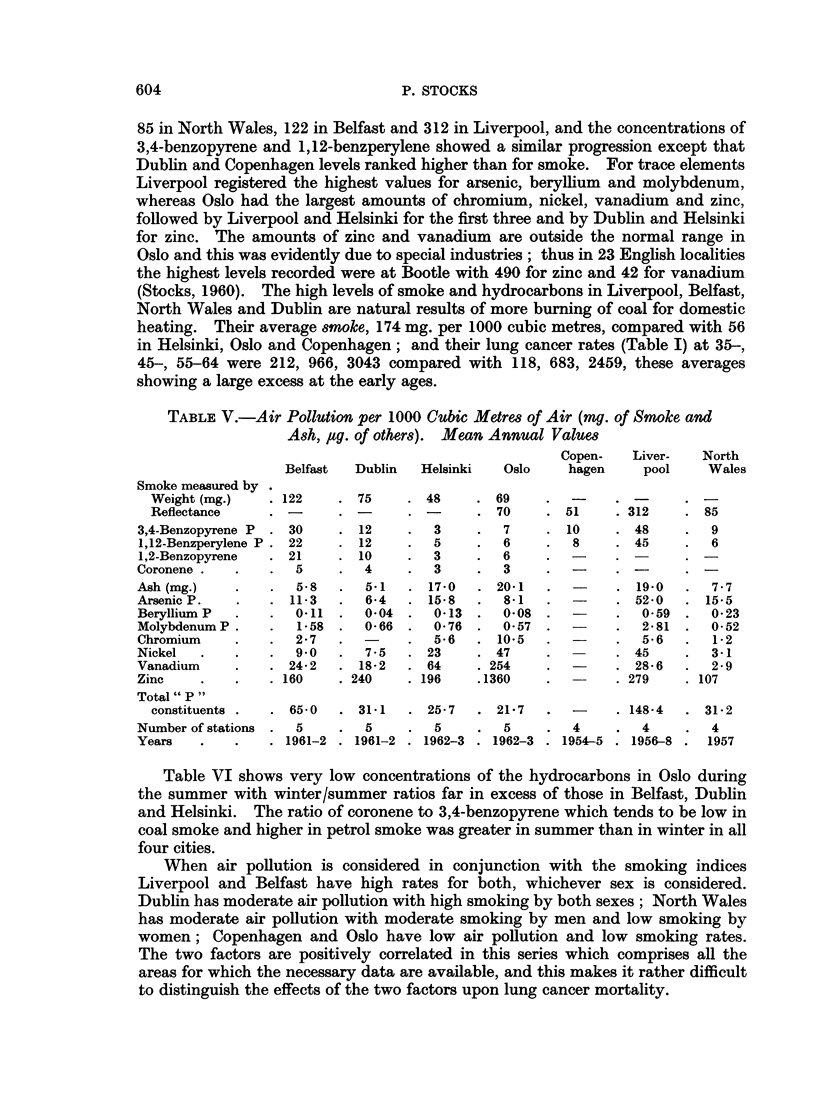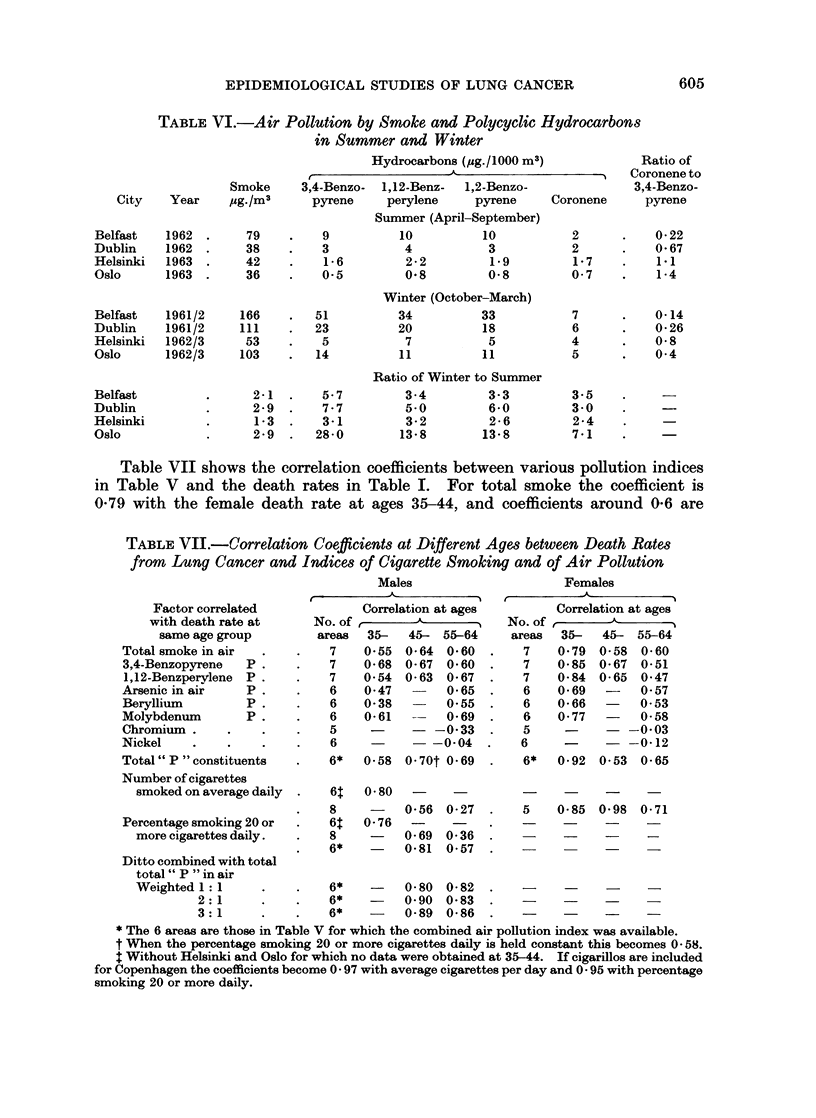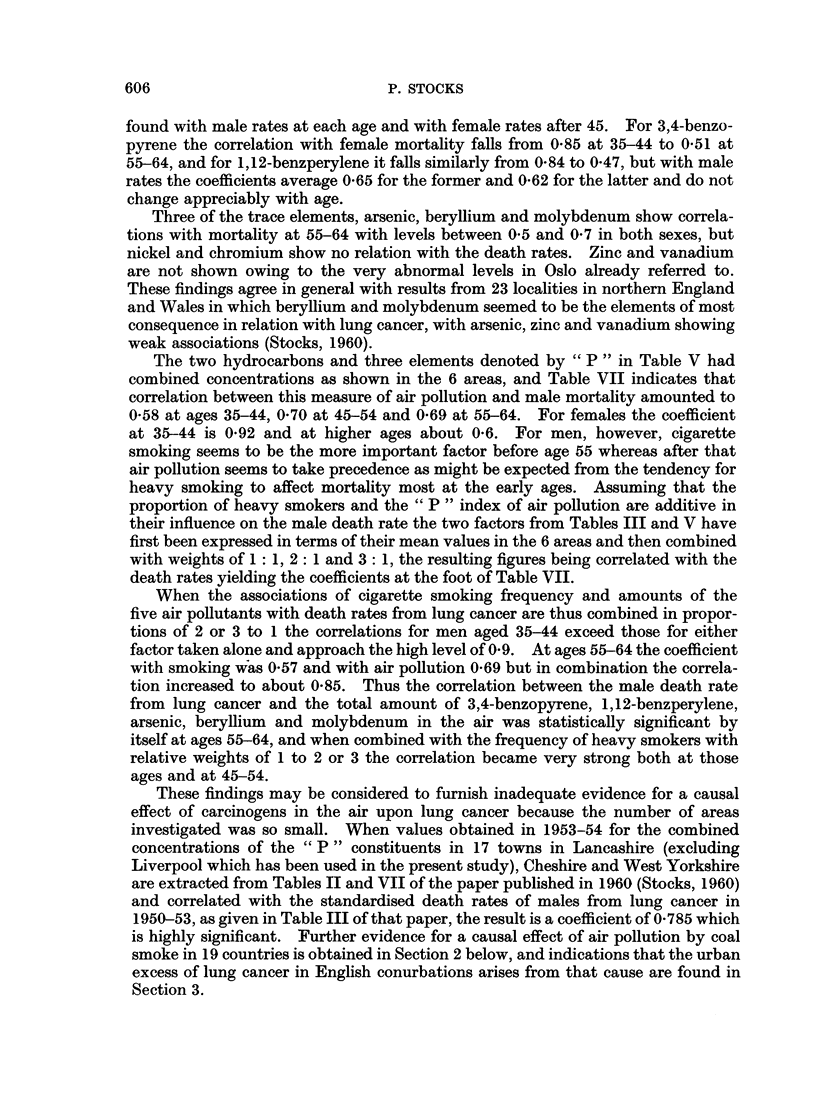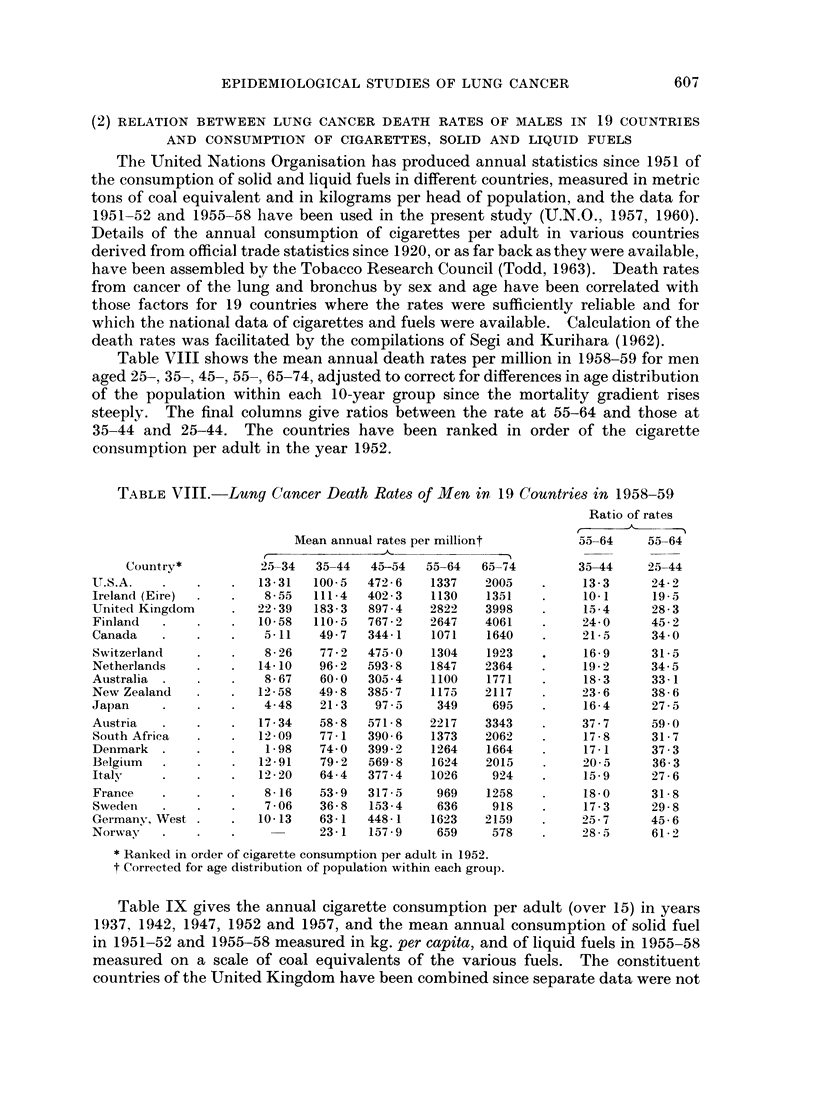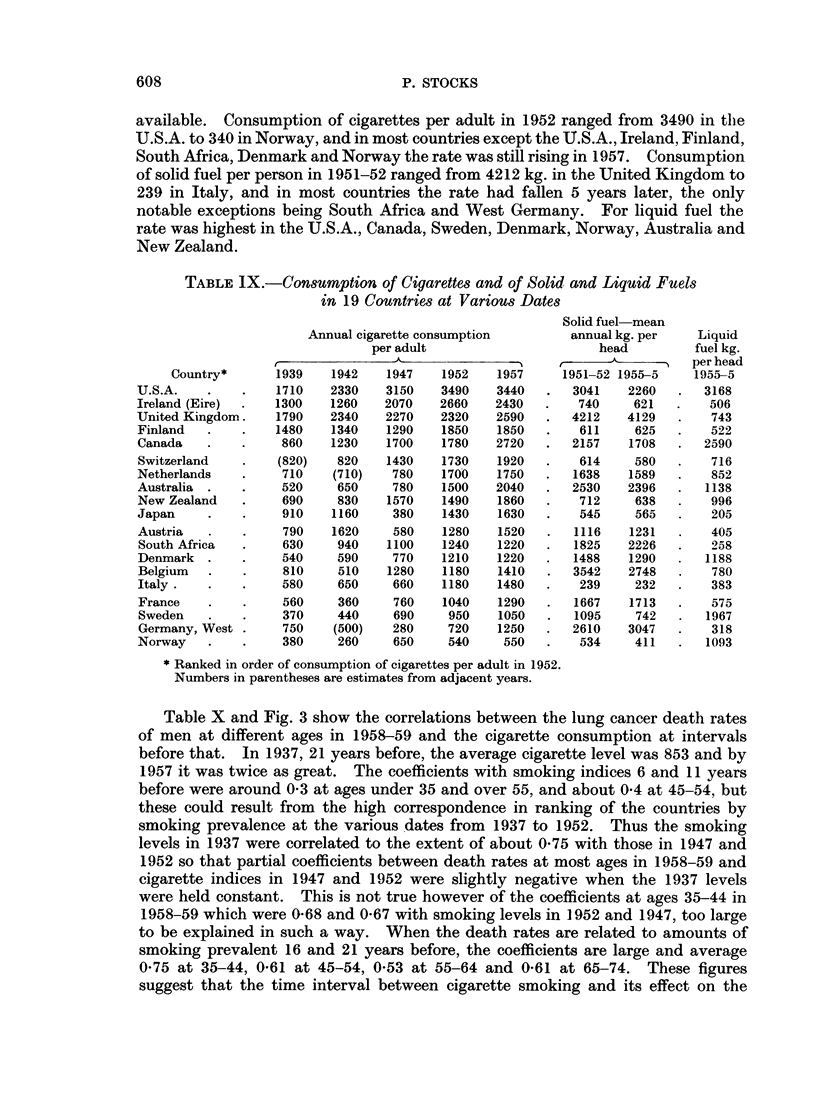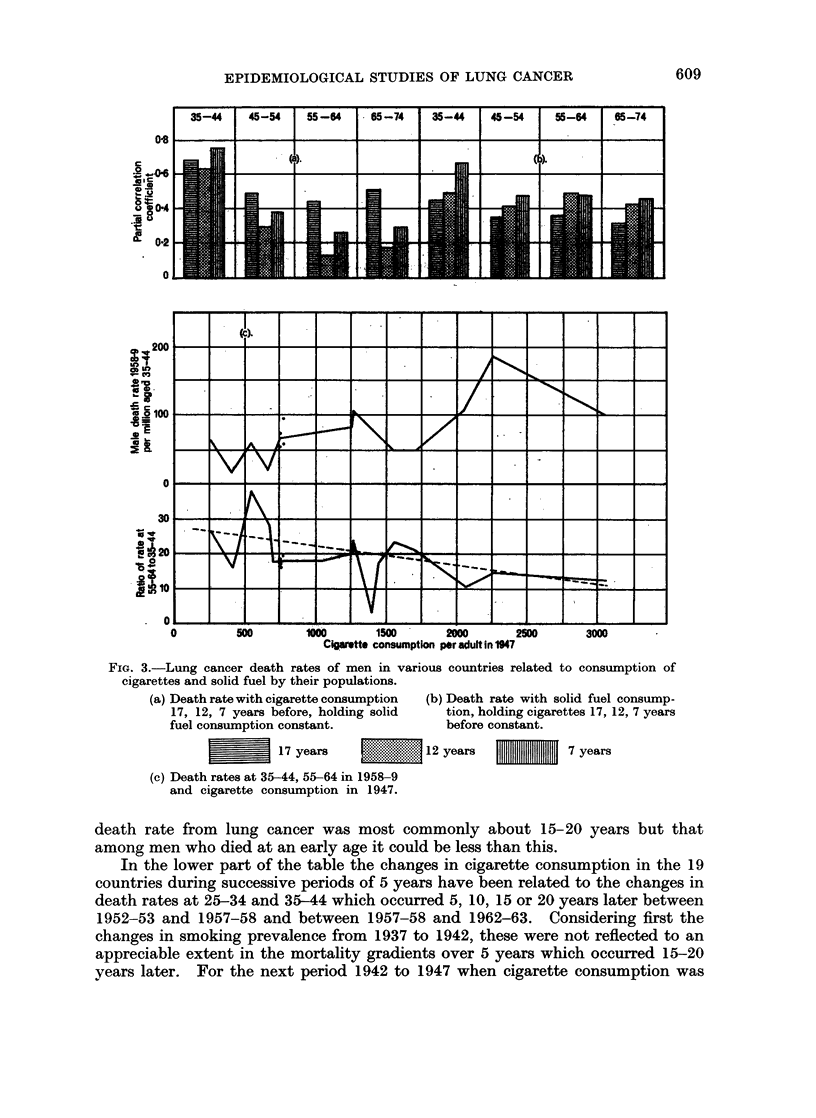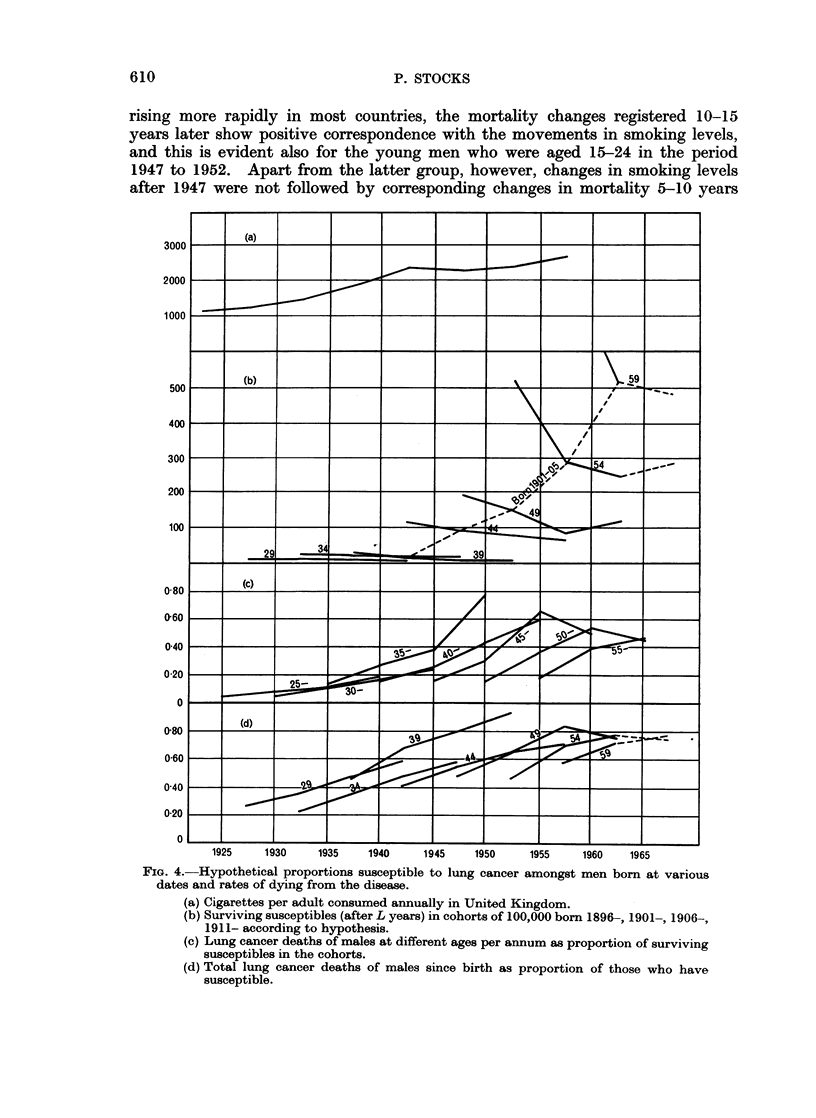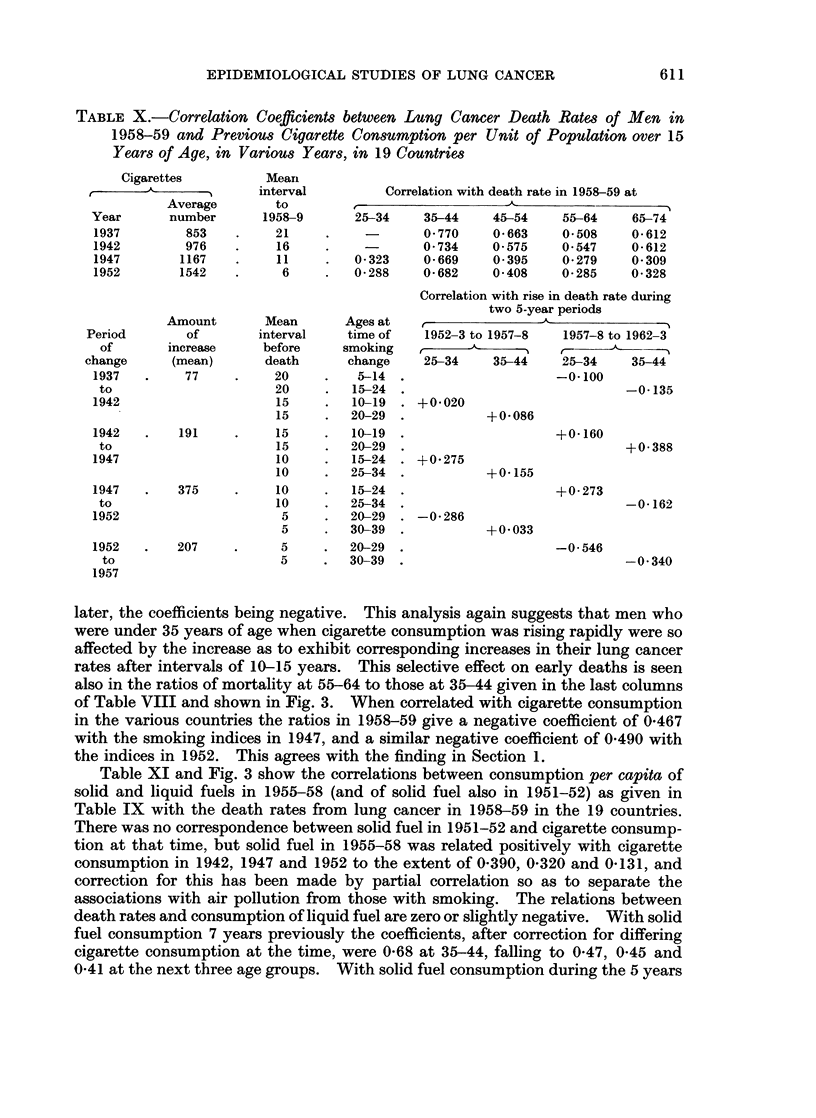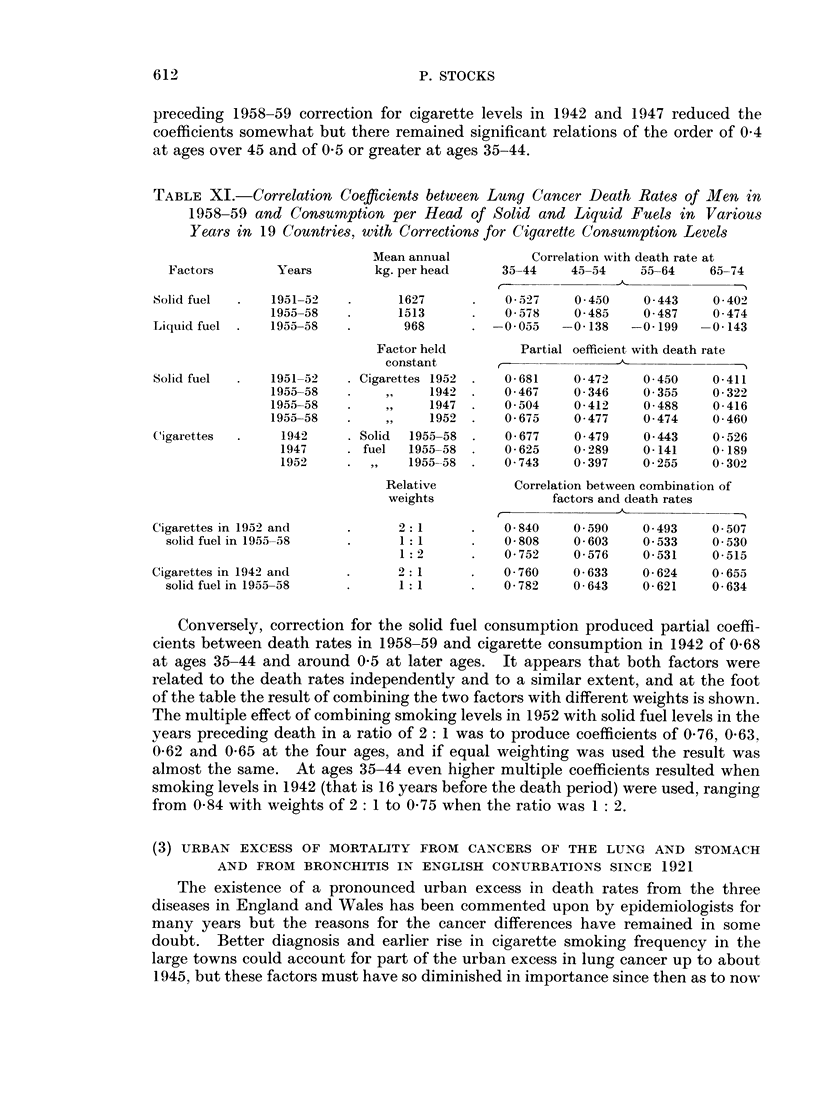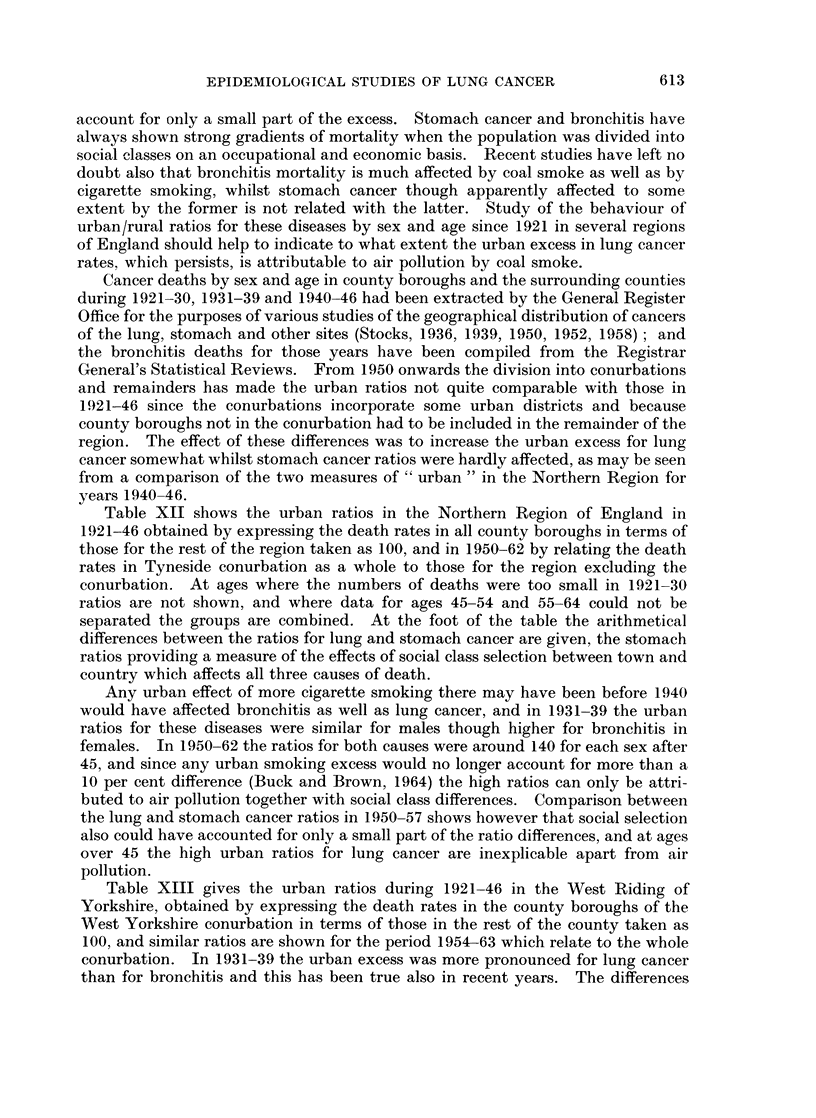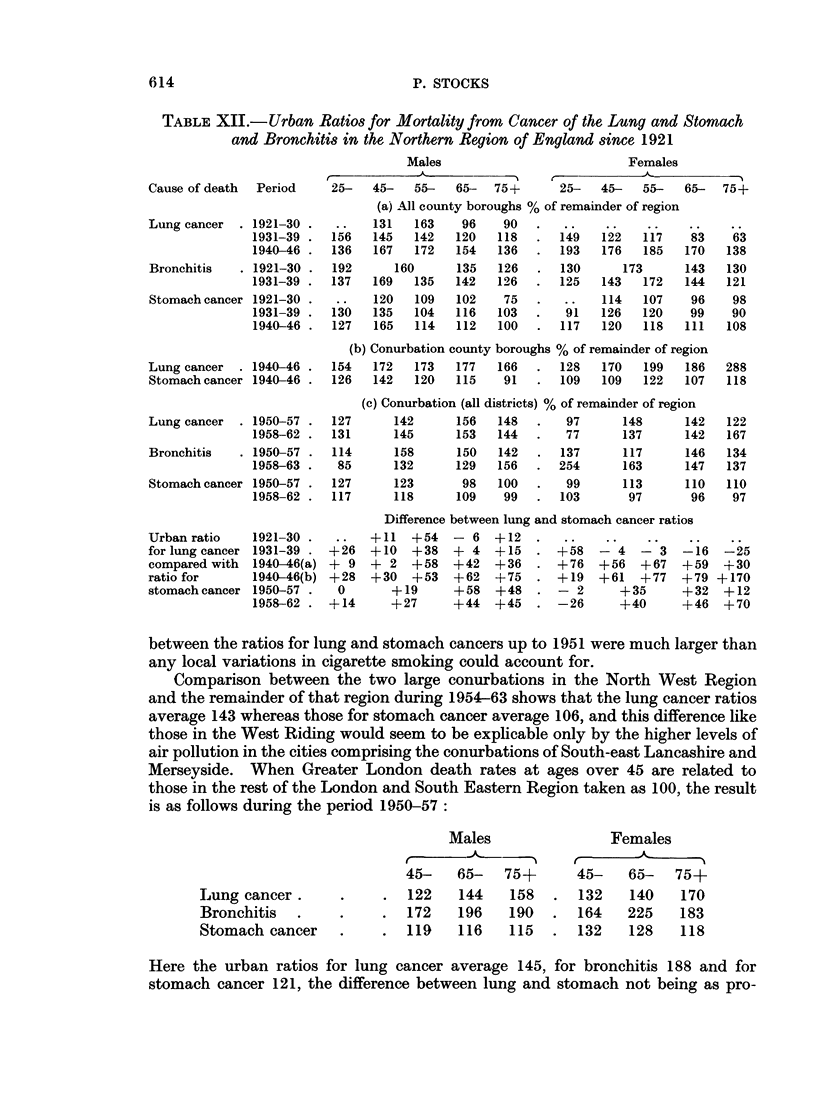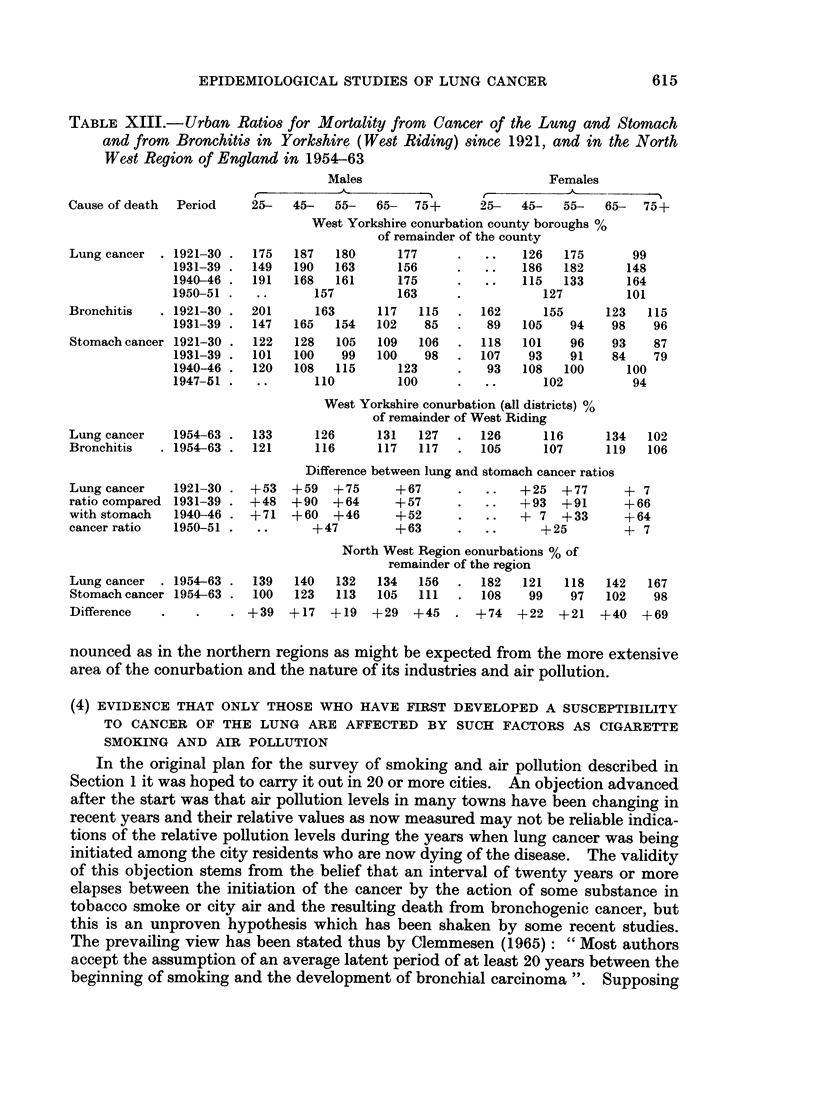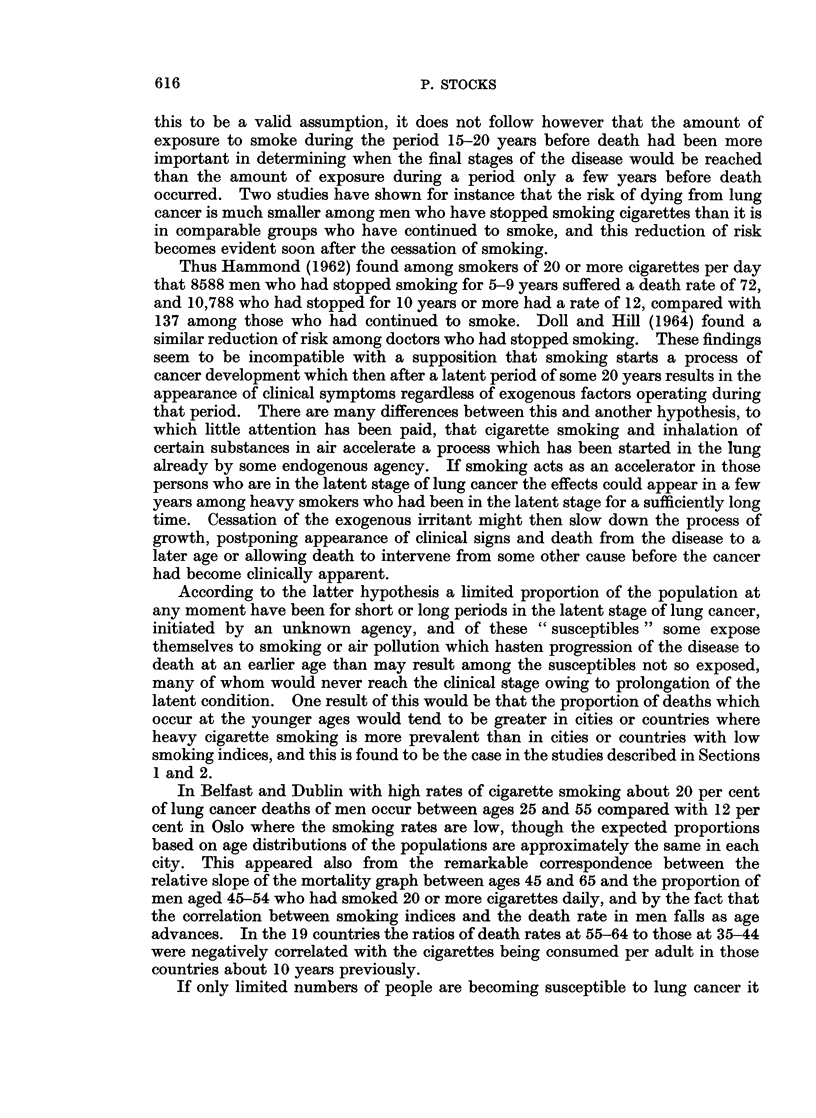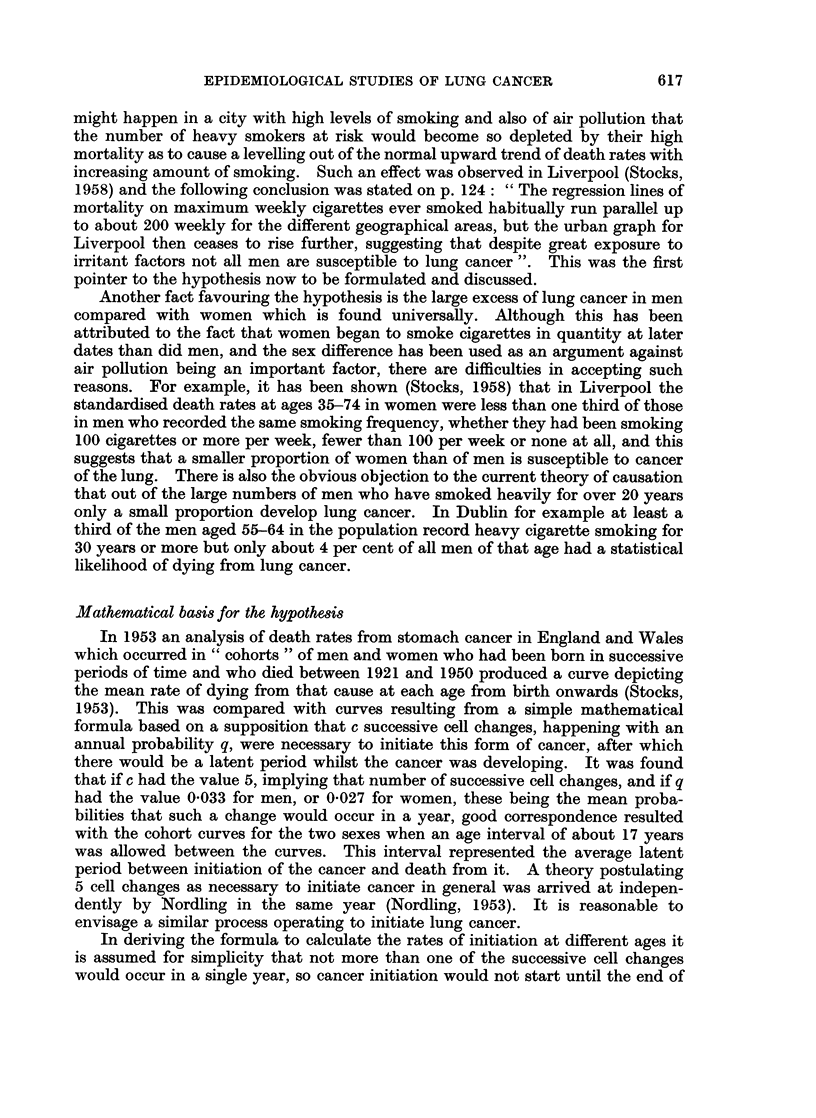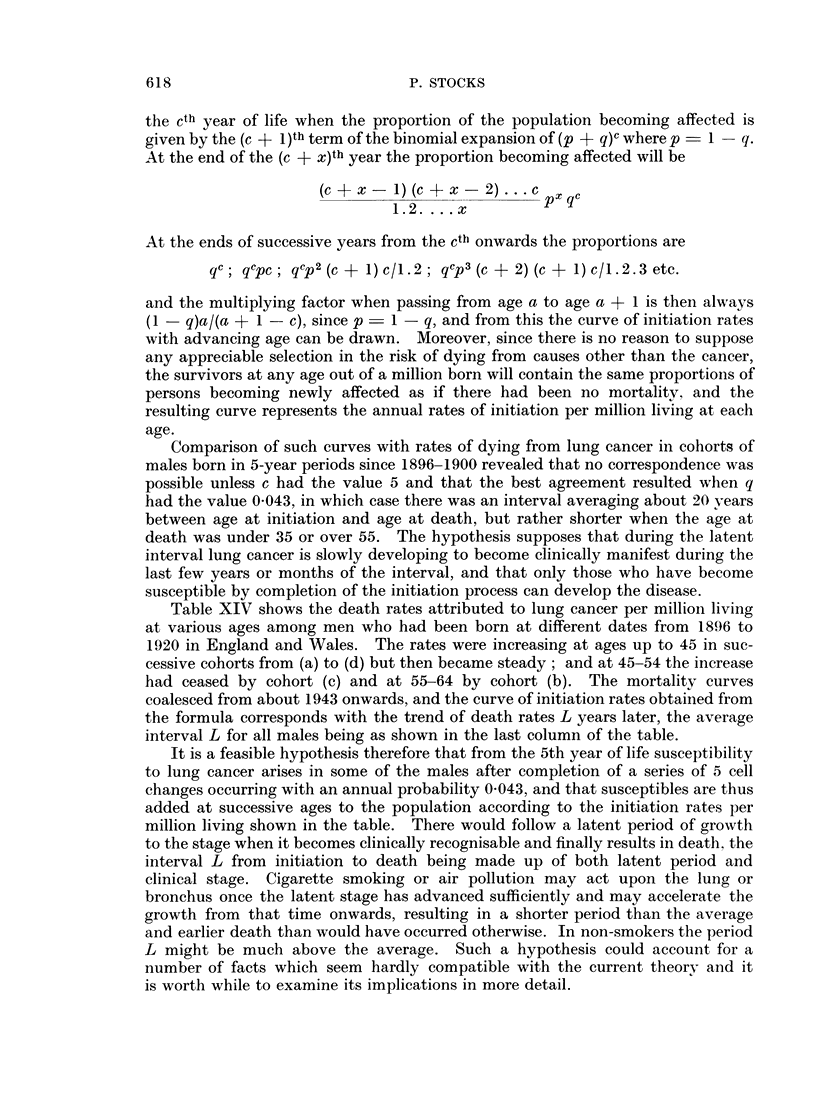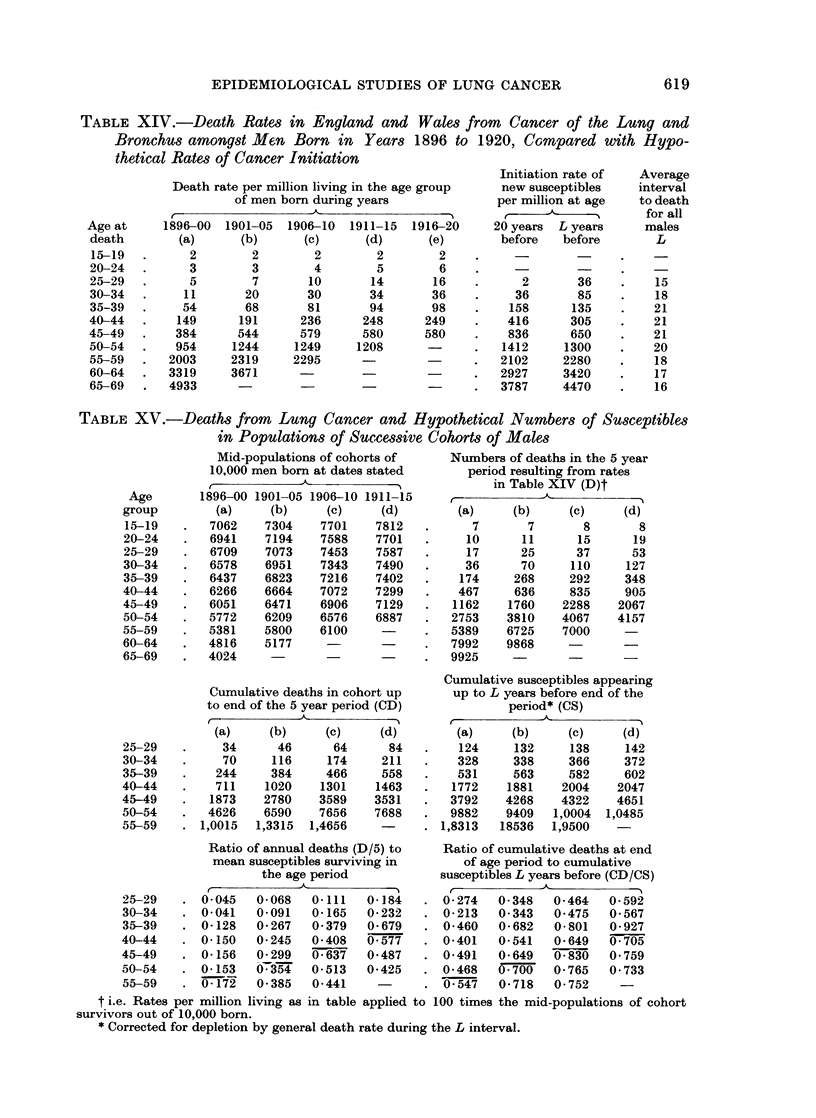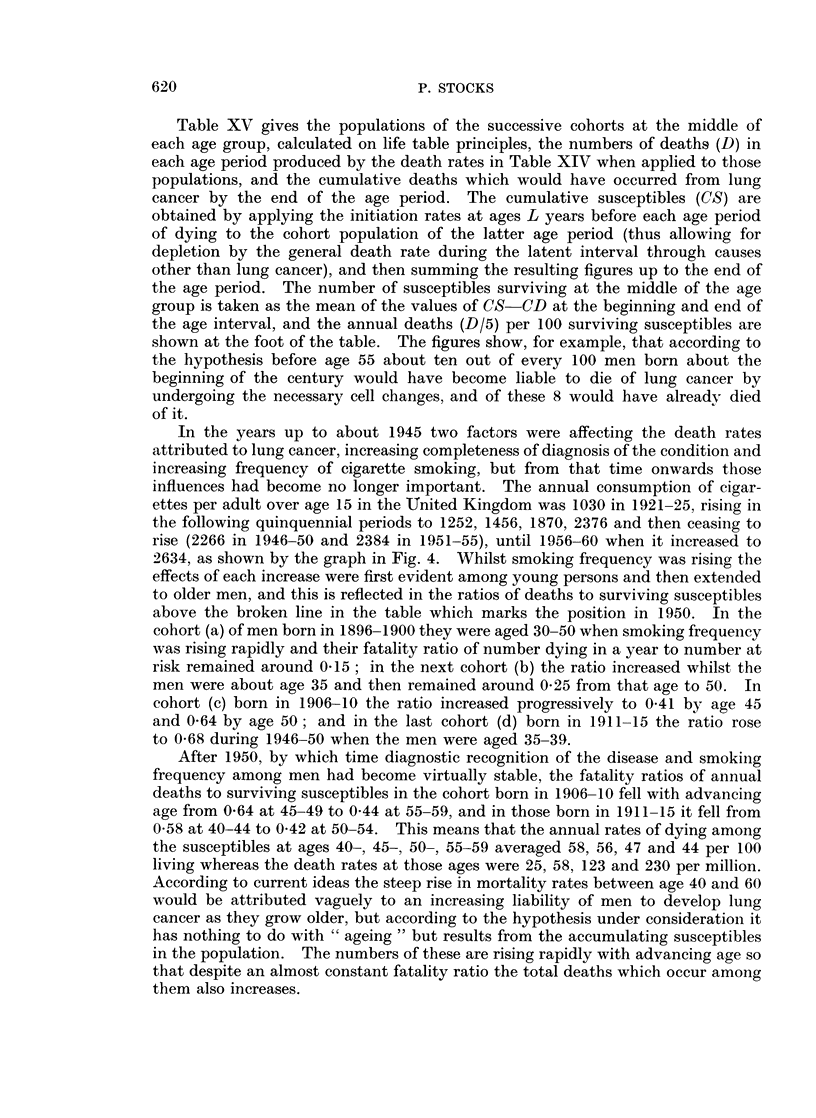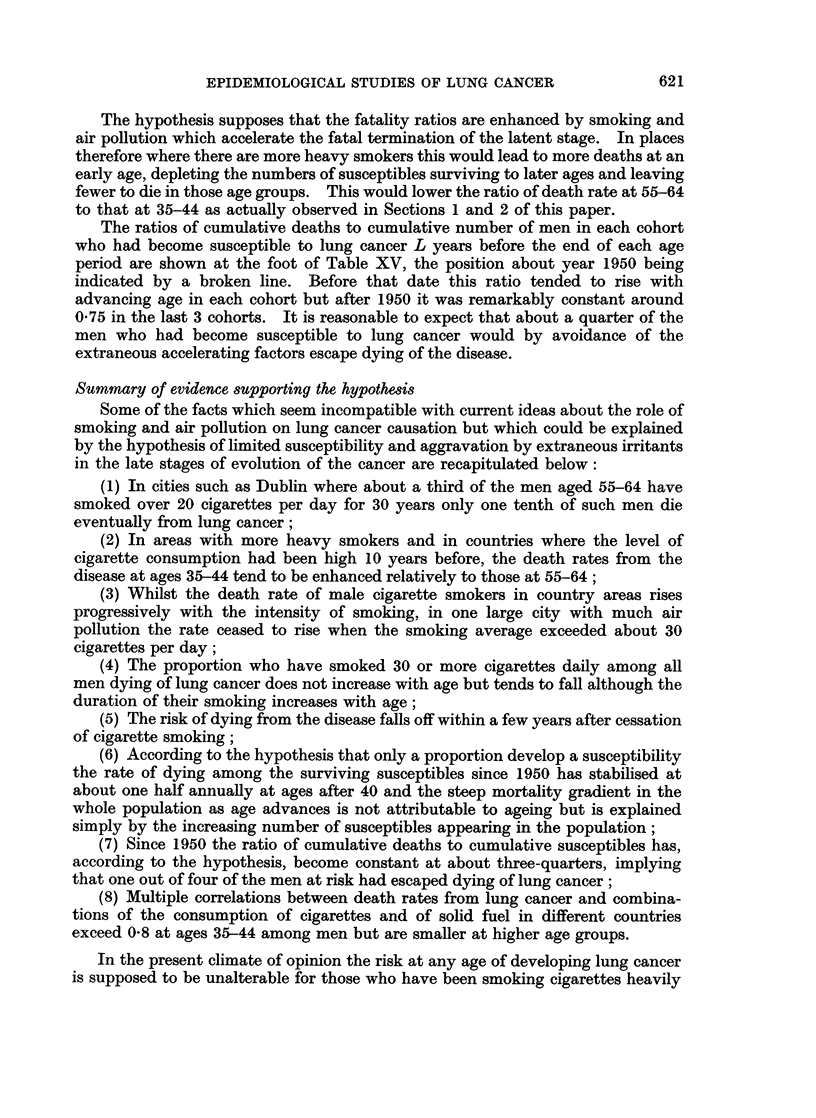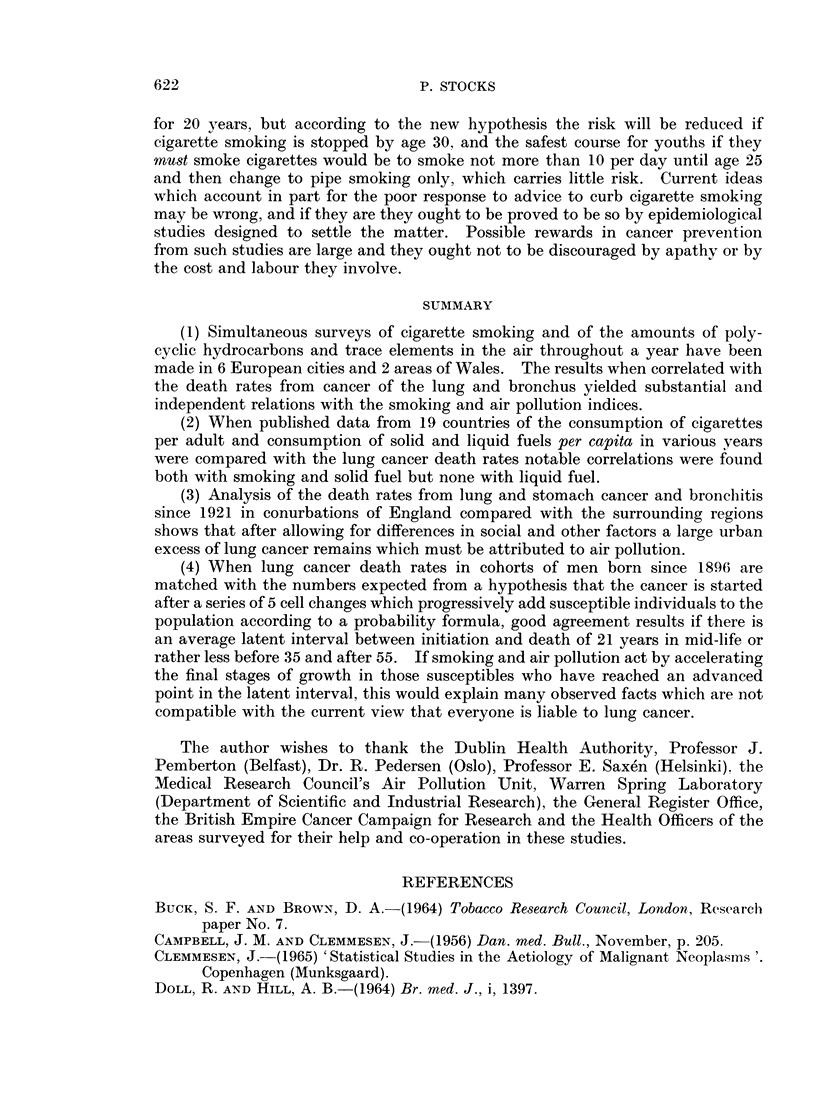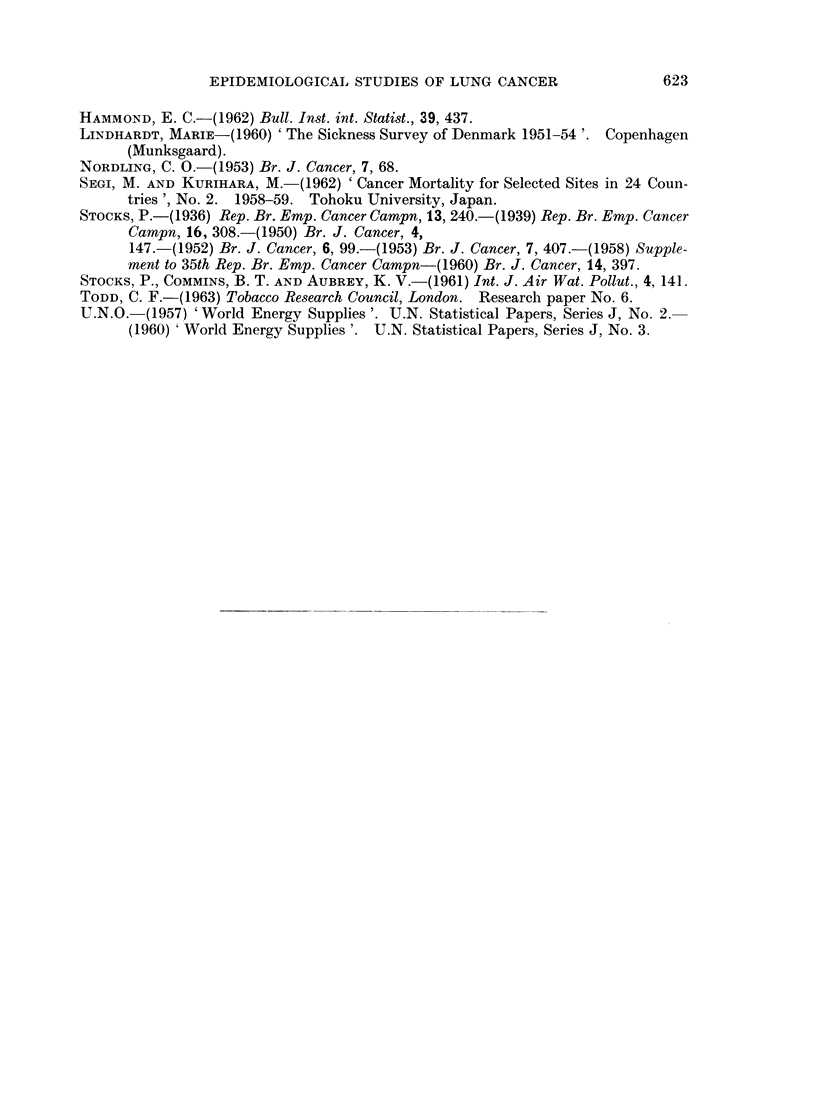# Recent epidemiological studies of lung cancer mortality cigarette smoking and air pollution, with discussion of a new hypothesis of causation.

**DOI:** 10.1038/bjc.1966.74

**Published:** 1966-12

**Authors:** P. Stocks


### BRITISH JOURNAL OF CANCER

VOL. XX DECEMBER, 1966 NO. 4

### RECENT EPIDEMIOLOGICAL STUDIES OF LUNG CANCER MORTALITY, CIGARETTE SMOKING AND AIR POLLUTION, WITH DISCUSSION OF A NEW HYPOTHESIS OF CAUSATION

P. STOCKS*

Received for publication August 3, 1966

IN 1960 the results of relating death rates from cancer of the lung and bronchus with measurements of air pollution by specific constituents of smoke obtained throughout a year in 23 districts in a geographical band from Anglesey to Tyneside in Britain were found to show substantial correlations after allowing for social factors (Stocks, 1960).

In the same year, following discussions on the epidemiology of cancer of the lung, a simultaneous study of smoking and air pollution was initiated in a number of cities and the outcome of this in conjunction with previous studies of a similar kind is recorded in Section 1.

Approaching the same problem from a different angle, a statistical study has been made of the cigarette consumption per adult and the consumption of solid and liquid fuels per head of population in 19 countries in various years related to lung cancer death rates of men at different ages, and the results of this are given in Section 2.

The outcome of an analysis of age-specific death rates from cancers of the lung and stomach in four conurbations of England and in the areas surrounding them during periods of years since 1921 with a view to a better understanding of the reasons for the urban excess in mortality is recorded in Section 3.

Finally, in Section 4, a hypothesis that smoking and air pollution affect only those persons who have first developed a susceptibility to lung cancer is examined in the light of the findings from these and other studies.

### (1) SURVEY OF CIGARETTE SMOKING, AIR POLLUTION AND LUNG CANCER MORTALITY IN EIGHT LOCALITIES

### *Initiation of the survey in four cities*

The survey of tobacco smoking and air pollution was made in four cities outside Great Britain where reliable death rates by age and cause were available. Histories of smoking were ascertained from samples of the population drawn from registers, and measurements of air pollution were made by a standard technique with analyses carried out at the same laboratory. The first cities investigated were Dublin and Belfast, and in 1961 Dr. J. B. O'Regan, Chief Medical Officer of the Dublin Health Authority, and his Deputy, Dr. M. Crowe, agreed to arrange in

* Address : 34 Brompton Avenue, Colwyn Bay, Denbighshire.

conjunction with the statistical office the selection of a sample of persons from the electoral register to produce about 2000 between ages 35 and 75, and to organise the interviewing at their homes by Health Visitors. They also helped to find 5 suitable sites for installation of air pollution instruments and undertook to arrange for regular changing of the filter papers throughout one year and for their despatch to the laboratory in London. Details of deaths from lung cancer and bronchitis by sex and age from 1955 onwards with the appropriate populations were provided by the statistical office. In Belfast similar arrangements were made with Professor J. Pemberton, Department of Social and Preventive Medicine, Queen's University, in conjunction with Dr. W. G. Swann, Medical Officer of Health, and the statistical office ; and as in Dublin, 5 sites were selected for the air filters. Dr. A. J. Tuyns helped in the local discussions.

Agreements were made with Warren Spring Laboratory of the Department of Scientific and Industrial Research, at Stevenage near London, for providing and installing the instruments to collect samples of suspended matter from the air and for a spectrographic analysis of the samples for certain trace elements, and with Dr. P. Lawther, Director of the Medical Research Council's Air Pollution Unit at St. Bartholomew's Hospital, London, for receiving the samples and analysing them for total smoke and certain polycyclic hydrocarbons.

The population surveys were carried out during 1961–62 and the filters began to operate in May 1962 and continued to do so without intermission night or day for 12 months so as to provide annual averages not influenced by seasonal variations. The questionnaires concerning smoking, residential and occupational histories were coded, transferred to punch cards and tabulated. Reports on this pilot study indicated that the methods which had been devised were satisfactory and could be repeated in other cities without change.

The procedure was repeated therefore in Helsinki and Oslo during 1962–63 as part of a special study of lung cancer in Finland and Norway. The original plan had envisaged the survey being made in about 20 cities, but because of certain difficulties this did not materialise and the data obtained from the 4 cities would have availed little had it not been that comparable data already existed for four other areas.

*Existing data from other localities.*

Studies had been published for several localities which satisfied the criteria required for comparability with the data from the four cities, the requirements being that (a) details of cigarette smoking in the population of various ages must be derived from large and valid samples, (b) air pollution must be measured throughout a year by filters at 4 or 5 points, (c) analyses of the suspended matter must be made at the same laboratories using the same technique, (d) reliable death rates from lung cancer must be available for a period centred about 1960 or 1961. Four areas have produced the necessary data to satisfy conditions (a) and (d), namely Copenhagen, Liverpool, North Wales excluding Wrexham, and the urban and rural districts of Wrexham ; and the first three of these satisfy conditions (b) and (c) by having air pollution data at 4 or 5 stations derived by the same methods and in the same laboratories as for Belfast, Dublin, Helsinki and Oslo.

Death rates from cancer of the lung and bronchus during 5 or 6 years in all the eight areas are shown by sex and age in Table I. At ages 35–44 for each sex Oslo has the lowest rate, followed by Copenhagen, and Liverpool has the highest, preceded by Dublin and Belfast. At each age group between 45 and 75, for men Oslo has the lowest rate followed by North Wales, whilst Liverpool has the highest, preceded by Helsinki; and for women Oslo, North Wales or Wrexham rank lowest with Liverpool or Dublin highest.

TABLE I.—*Death Rates per Million from Cancer of the Lung and Bronchus* (*International No.* 162, 163)

| Age group | Belfast | Dublin | Helsinki | Oslo | Copen-hagen | Liver-pool | North Wales* | Wrexham† |
|---|---|---|---|---|---|---|---|---|
| *Males* | | | | | | | | |
| 25–34 | 21 | 26 | 0 | 0 | 40 | 48 | 0 | 0 |
| 35–44 | 225 | 234 | 211 | 41 | 103 | 263 | 127 | 127 |
| 45–54 | 936 | 949 | 1088 | 298 | 663 | 1364 | 616 | 669 |
| 55–64 | 2821 | 3124 | 3238 | 1368 | 2770 | 3986 | 2242 | 2506 |
| 65–74 | 4243 | 4351 | 5080 | 1973 | (4600) | 6510 | 3478 | 4455 |
| 75 and over | 2652 | 2788 | 4846 | 1537 | | 4534 | 2037 | 3186 |
| *Females* | | | | | | | | |
| 25–34 | 20 | 9 | 0 | 0 | 0 | 22 | 0 | 0 |
| 35–44 | 63 | 75 | 34 | 0 | 30 | 110 | 46 | 49 |
| 45–54 | 178 | 229 | 98 | 75 | 117 | 201 | 86 | 99 |
| 55–64 | 213 | 394 | 200 | 182 | 307 | 420 | 210 | 169 |
| 65–74 | 437 | 593 | 326 | 280 | (480) | 620 | 274 | 287 |
| 75 and over | 557 | 964 | 485 | 352 | | 773 | 353 | 289 |
| Years | 1958–63 | 1958–63 | 1959–63 | 1959–63 | 1958–62 | 1958–63 | 1958–63 | 1958–63 |

* Registrar General's Wales II region except Wrexham area.
† Wrexham Municipal Borough and Rural District.

### *Population samples for interview*

*Belfast.*—The electoral register in 1960 contained 274,450 names, corresponding approximately with the population aged 22 and over. A sample of 3550 was drawn by a random technique of whom 160 were found to reside outside the city, 58 had died, 138 were voters on account of business premises and duplicated, 134 could not be interviewed and 51 refused to be questioned. The age distributions of the remaining 926 men and 1188 women with ages between 35 and 75 are shown in Table II. Interviews were carried out by persons specially trained for the work.

TABLE II.—*Sex and Age Distribution of the Population Samples*

| Age group | Number of persons interviewed | | | | | |
|---|---|---|---|---|---|---|
| | Belfast | | Dublin | | Helsinki | Oslo |
| | Males | Females | Males | Females | Males | Males |
| 35–44 | 277 | 362 | 276 | 366 | — | — |
| 45–54 | 283 | 334 | 269 | 345 | 303 | 481 |
| 55–64 | 226 | 306 | 237 | 233 | 687 | 856 |
| 65–74 | 140 | 186 | 127 | 146 | — | — |
| *Totals* | | | | | | |
| 35–74 | 926 | 1188 | 909 | 1090 | — | — |
| 45–64 | — | — | — | — | 990 | 1337 |
| *Subgroups* | | | | | | |
| 55–59 | — | — | — | — | 331 | 419 |
| 60–64 | — | — | — | — | 356 | 437 |

*Dublin*.—The sampling procedure was similar to that in Belfast, giving 909 men and 1090 women of the required ages, and the interviews were effected by trained personnel on the staff of the Health Authority.

*Helsinki and Oslo*.—A comparative study of lung cancer in Finland and Norway began in 1962, and I was permitted to use tabulated data of cigarette smoking amongst men aged 45–54, 55–59 and 60–64 resident in the two capital cities, prepared by Dr. E. Pedersen at Oslo in conjunction with Professor E. Saxén at Helsinki. Interviews were carried out by public health nurses who had been trained for the survey. In Oslo 1337 and in Helsinki 990 men were interviewed as to their smoking habits with ages as shown in Table II.

*Copenhagen*.—In the course of a morbidity survey of Denmark in 1951–54 smoking histories were obtained from representative samples of 3277 men and 3697 women in the city, details being published in a report by Lindhardt (1960). Approximately every thousandth card was drawn from the population register at monthly intervals and the age distribution was checked against that of the whole population. The questions asked of the 84 per cent of persons successfully interviewed covered weekly consumption of cigarettes, cigarillos, cigars and grams of pipe tobacco. Alternative smoking rates which included cigarillos along with cigarettes were given and have been taken account of in this paper, and since total cigarette consumption according to trade statistics exceeded that calculated from the interviews, correction for understatement has been made in the Copenhagen estimates of numbers of cigarettes smoked.

*Liverpool*.—In a cancer survey during 1953–55 all adults admitted as inpatients to a large general hospital in the city, numbering 3353 at ages 35–74, were interviewed and questioned as to their histories of residence, occupations and smoking, and the details of smoking by men and women were published (Stocks, 1958). To allow for a possible tendency for heavy smokers to form a larger proportion of hospital inpatients than would be found in the general population of the same ages, smoking rates among some 360 men who died from cancer of the stomach, a condition not apparently affected by cigarettes, were used as a check. For men, but not women, hospital selection was significant and the average numbers of cigarettes smoked per day shown in Table III of this report have been adjusted to allow for it by factors 0·90, 0·75, 0·80 at ages 35–54, 55–64 and 65–74.

*North Wales and Wrexham*.—The survey of 1953–55 covered these areas and all adult inpatients admitted to the general hospitals at Wrexham, Bangor, Llandudno and Rhyl were questioned as to their smoking records as in Liverpool. The results for 4637 persons in the larger area and for 1201 men in Wrexham districts were given by age groups in Tables 31 and 32 of the survey report (Stocks, 1958), and no correction was found necessary for hospital selection in these areas.

*Measurement of air pollution.*

The equipment was made by the Warren Spring Laboratory and consisted of an inverted funnel fixed outside a window at 3 to 6 metres above ground level with inlet tube dividing into two channels, each leading through a filter, suction pump and gas meter to measure the volume of air drawn in, which averaged 4 cubic metres per day. One filter of glass fibre was changed monthly and the other of paper every 3 months, deposits being analysed respectively by the Medical Research Council's Air Pollution Unit in London and the Warren Spring Laboratory. At Belfast and Dublin the changing of filters was done by the Health

Departments, in Oslo it was directed by the Institute of Hygiene (Professor Nutting) and in Helsinki by the Institute of Occupational Health (Professor Nord). The apparatus and techniques used in Liverpool and North Wales had been virtually the same as above, but in Copenhagen trace elements were not analysed so only one filter was used at each site.

When suspended matter is collected in this way by continuous passage of air, variations due to weather and season are mostly eliminated but concentrations at different points in a city may differ, and in order to reduce this variability 4 or 5 sites were chosen so that the average of the measurements would give as good an index as possible of the pollution to which a resident was exposed during a year. In North Wales the sites were in the Conway Valley, Llangefni, Ruthin and Flint, the first of these being rural and the others small towns typical of the area. In Belfast, Dublin, Helsinki and Oslo amounts of total smoke were found by direct weighing of the deposit on the glass fibre filters whilst in the earlier studies the reflection method of estimation had been used (Stocks, 1960). In Oslo the two measures differed by only $1\frac{1}{2}$ per cent but in Dublin and Belfast rather larger differences were found.

The polycyclic hydrocarbons determined were 3,4-benzopyrene, 1,12-benzperylene, 1,2-benzopyrene and coronene, and the analyses were made by the technique described by Stocks, Commins and Aubrey (1961). The trace elements shown in Table IV were determined at Warren Spring Laboratory by spectrographic methods as described in the same paper, the filters being aggregated for each site for a whole year so as to produce sufficient material. For the hydrocarbons six month aggregates were examined, designated summer and winter in Table VI.

In Copenhagen during a year starting in October 1954, sampling stations had been installed at four sites, the filter papers being analysed in London and the results published by Campbell and Clemmesen (1956). In Liverpool and North Wales 4 sampling stations were used in each area from October 1954, and the results for total smoke and a number of hydrocarbons and trace elements were published in a series of papers (Stocks, 1958, 1960 ; Stocks *et al.*, 1961).

*Cigarette smoking and lung cancer mortality*

Table III shows the indices of cigarette smoking according to three measures, namely : (1) proportion per 100 persons interviewed who had been habitual smokers of cigarettes at any time, (2) average number of cigarettes smoked per day in the present, or past if no longer smoking, per unit of population, (3) proportion per 100 population who had been smokers of 20 or more cigarettes per day. Gaps in the table indicate that information was not available or that rates could not be properly evaluated.

The proportion of men who had smoked cigarettes ranged from about 80 per cent in Helsinki and Wrexham to 94 per cent in Liverpool at ages 45–54. Among women the proportions diminished rapidly with advancing age. The average number smoked per day by men aged 45–54 ranged from over 18 in Liverpool, Belfast and Dublin to 13 in Oslo and 7 (or 12 if cigarillos are included) in Copenhagen. The relations with death rates are seen in Fig. 1.

Table VII shows that when the average number of cigarettes per day is correlated with the death rate of the same age group the coefficients for women are 0·85 at 35–44, 0·98 at 45–54 and 0·71 at 55–64, whereas the coefficient for men falls from 0·80 at 35–44 (or 0·97 if cigarillos are included in the Copenhagen

TABLE III.—*Tobacco Smoking Histories of Population Samples by Sex and Age, relating to Cigarettes*

| | Males | | | | | Females | | | |
|---|---|---|---|---|---|---|---|---|---|
| | 35– | 45– | 55– | 65–74 | | 35– | 45– | 55– | 65–74 |
| | Percentage of population who have been smokers of cigarettes habitually at any time | | | | | | | | |
| Belfast | 80·7 | 86·5 | 85·5 | 70·4 | . | 52·2 | 41·9 | 24·9 | 16·1 |
| Dublin | 82·4 | 85·9 | 86·7 | 68·3 | . | 55·5 | 54·0 | 40·8 | 28·8 |
| Helsinki | — | 80·5 | 79·9 | — | . | — | — | — | — |
| Oslo | — | 84·0 | 77·7 | — | . | — | — | — | — |
| Copenhagen | — | — | — | — | . | 46 | 30 | 17 | 9 |
| Liverpool | 89·0 | 93·7 | 87·1 | 80·7 | . | 58·9 | 48·6 | 31·4 | 23·7 |
| North Wales | 80·4 | 85·4 | 77·5 | 58·0 | . | 31·0 | 21·4 | 17·0 | 5·4 |
| Wrexham | 79·3 | 79·6 | 75·5 | 55·4 | . | — | — | — | — |
| | Average number of cigarettes smoked per day by present or past smokers, per unit of population | | | | | | | | |
| Belfast | 16·4 | 18·2 | 16·3 | 13·4 | . | 6·4 | 5·4 | 2·7 | 2·0 |
| Dublin | 16·5 | 18·2 | 17·7 | 13·2 | . | 7·8 | 7·7 | 6·6 | 3·2 |
| Helsinki | — | 16·2 | 12·7 | — | . | — | — | — | — |
| Oslo | — | 12·6 | 10·5 | — | . | — | — | — | — |
| Copenhagen* | 8·9 | 6·8 | 3·8 | 1·1 | . | 4·3 | 2·8 | 1·4 | 0·6 |
| Liverpool | 16·8 | 18·6 | 14·7 | 14·2 | . | 8·8 | 7·1 | 4·1 | 1·8 |
| North Wales | 14·8 | 16·5 | 14·6 | 10·0 | . | 3·3 | 2·2 | 1·8 | 0·6 |
| Wrexham | 14·6 | 15·4 | 13·7 | 9·1 | . | — | — | — | — |
| | Percentage of population who had been smokers of 20 or more cigarettes per day | | | | | | | | |
| Belfast | 36·7 | 50·7 | 40·7 | 26 | . | 7·7 | 8·1 | 3·9 | 3·2 |
| Dublin | 40·3 | 48·7 | 45·5 | 34·9 | . | 14·6 | 11·5 | 14·6 | 4·1 |
| Helsinki | — | 42·2 | 31·5 | — | . | — | — | — | — |
| Oslo | — | 24·4 | 16·9 | — | . | — | — | — | — |
| Copenhagen† | 15 | 11 | 6 | 1 | . | — | — | — | — |
| Liverpool | 37·6 | 45·9 | 30·1 | 27·2 | . | 16·3 | 13·7 | 7·6 | 1·0 |
| North Wales | 27·9 | 33·3 | 27·4 | 19·9 | . | 4·5 | 3·2 | 2·8 | 0·8 |
| Wrexham | 25·8 | 31·3 | 25·4 | 14·6 | . | — | — | — | — |

* After applying the correction factor for under-statement as calculated from total cigarette sales. If cigarillos and cigars are added the average numbers for males are raised to 13·2, 12·2, 13·0, 8·7.

† After correcting for under-statement. If cigarillos and cigars are added the percentages smoking 20 or more per day are raised to 21, 20, 21, 8.

average) to 0·56 at 45–54 and 0·27 at 55–64, the figure at 65–74 which does not appear in the table being 0·24.

The third measure of smoking, proportion of all men of the specified ages who had smoked habitually 20 or more cigarettes daily, is seen from Table III and Fig. 1 to have exceeded 45 per cent in Belfast, Dublin and Liverpool at ages 45–54 and to have exceeded 40 per cent in the first two of these cities at 55–64, compared with levels below 25 per cent in Oslo and Copenhagen. Table VII shows that when this heavy smoking index is correlated with the death rate at the same age the coefficients are 0·76 at 35–44 (or 0·95 if cigarillos are included in Copenhagen), 0·69 at 45–54 and 0·36 at 55–64. It appears from the two measures of amounts smoked that the effect of excessive cigarette smoking in raising death rates from lung cancer is greatest around age 40 and becomes progressively weaker at later ages.

In Fig. 2 the differential effect according to age is shown by the convergence of the graphs of the logarithms of the death rates at 35–44, 45–54 and 55–64, but not

FIG. 1.—Average number of cigarettes which had been smoked per day by all men and women aged 35–44, 45–54 and 55–64, and proportion who had smoked 20 or more daily in 8 areas arranged from left to right in order of increasing death rate from lung cancer at ages 45–54. Cigarettes daily (columns) :

Ages 35–44   45–54   55–64

—— Percentage who smoked 20 or more daily (graph).

of those at 55–64 and 65–74, when they are plotted against the proportion of men aged 45–54 who had smoked 20 or more cigarettes per day in the upper diagram, and against the average number smoked per day in the lower diagram. Logarithms have been used for the vertical scale because the death rates increase so greatly from 35 to 75 and because the distance between the graphs is a measure of the ratio between the death rates at that point. Whichever index of smoking is used the separation between the graphs at 35–44 and 55–64 decreases as the index rises, and this is shown at the foot of each diagram by graphs depicting the trend of the logarithm of the ratio between mortality at 55–64 and 35–44. The actual ratios are given in Table IV where the localities are arranged from left to right in descending order of the proportion of men aged 45–54 who had been smokers of 20 or more cigarettes daily. The ratio between the death rates at 55–64 and 35–44 rises without intermission from 12·5 to 33·3 as the smoking index falls from 18·2 to 12·6 per cent, and the correlation coefficient is 0·87. Correspondence with the other smoking index is almost as good. In contrast the slope of the mortality curve after 55 as indicated by the ratios does not correspond with smoking frequencies.

TABLE IV.—*Relation between Lung Cancer Mortality and Age in Men Compared with Amounts of Cigarette Smoking*

| | Belfast | Dublin | Liver-pool | Hel-sinki | North Wales | Wrex-ham | Oslo |
|---|---|---|---|---|---|---|---|
| Logarithm of death rates at ages : | | | | | | | |
| 35–44 | 2·352 | 2·369 | 2·420 | 2·324 | 2·104 | 2·104 | 1·613 |
| 45–54 | 2·971 | 2·977 | 3·135 | 3·037 | 2·790 | 2·825 | 2·474 |
| 55–64 | 3·450 | 3·495 | 3·601 | 3·510 | 3·351 | 3·399 | 3·136 |
| 65–74 | 3·628 | 3·639 | 3·814 | 3·706 | 3·541 | 3·649 | 3·295 |
| Ratio between rates (by log difference) | | | | | | | |
| 45–54/35–44 | 4·16 | 4·06 | 5·19 | 5·16 | 4·85 | 5·27 | 7·27 |
| 55–64/45–54 | 3·01 | 3·29 | 2·92 | 2·98 | 3·64 | 3·74 | 4·58 |
| 65–74/55–64 | 1·50 | 1·39 | 1·63 | 1·57 | 1·55 | 1·78 | 1·44 |
| 55–64/35–44 | 12·5 | 13·4 | 15·2 | 15·3 | 17·7 | 19·7 | 33·3 |
| Average cigarettes per day at ages : | | | | | | | |
| 45–64 | 18·2 | 18·2 | 18·6 | 16·2 | 16·5 | 15·4 | 12·6 |
| 55–64 | 16·3 | 17·7 | 14·7 | 12·7 | 14·6 | 13·7 | 10·5 |
| Percentage smoking 20 or more daily | | | | | | | |
| 45–54 | 50·7 | 48·7 | 45·9 | 42·2 | 33·3 | 31·3 | 24·4 |
| 55–64 | 40·7 | 45·5 | 30·1 | 31·5 | 27·4 | 25·4 | 16·9 |

This diminishing relation with smoking indices in men as age advances and the disproportionate enhancement of mortality about age 40 where smoking indices are high is incomprehensible according to current ideas as to how smoking affects lung cancer incidence, and this will be discussed further in Sections 2 and 4.

*Air pollution and lung cancer mortality*

Table V gives the mean annual concentrations in air of total suspended matter and mineral ash, 4 polycyclic hydrocarbons and 7 trace elements collected throughout a year at the filter stations in each area. The total smoke ranged from about 50 mg. per cubic metre in Helsinki and Copenhagen to 70 in Oslo, 75 in Dublin,

FIG. 2.—Correlation of logarithms of death rates from lung cancer at different ages with average numbers of cigarettes which had been smoked per day and proportion who had smoked 20 or more daily among all men aged 45–54 in 8 areas.

85 in North Wales, 122 in Belfast and 312 in Liverpool, and the concentrations of 3,4-benzopyrene and 1,12-benzperylene showed a similar progression except that Dublin and Copenhagen levels ranked higher than for smoke. For trace elements Liverpool registered the highest values for arsenic, beryllium and molybdenum, whereas Oslo had the largest amounts of chromium, nickel, vanadium and zinc, followed by Liverpool and Helsinki for the first three and by Dublin and Helsinki for zinc. The amounts of zinc and vanadium are outside the normal range in Oslo and this was evidently due to special industries ; thus in 23 English localities the highest levels recorded were at Bootle with 490 for zinc and 42 for vanadium (Stocks, 1960). The high levels of smoke and hydrocarbons in Liverpool, Belfast, North Wales and Dublin are natural results of more burning of coal for domestic heating. Their average *smoke*, 174 mg. per 1000 cubic metres, compared with 56 in Helsinki, Oslo and Copenhagen ; and their lung cancer rates (Table I) at 35–, 45–, 55–64 were 212, 966, 3043 compared with 118, 683, 2459, these averages showing a large excess at the early ages.

TABLE V.—*Air Pollution per* 1000 *Cubic Metres of Air* (*mg. of Smoke and Ash, μg. of others*). *Mean Annual Values*

| | Belfast | Dublin | Helsinki | Oslo | Copenhagen | Liverpool | North Wales |
|---|---|---|---|---|---|---|---|
| Smoke measured by | | | | | | | |
| Weight (mg.) | 122 | 75 | 48 | 69 | — | — | — |
| Reflectance | — | — | — | 70 | 51 | 312 | 85 |
| 3,4-Benzopyrene P | 30 | 12 | 3 | 7 | 10 | 48 | 9 |
| 1,12-Benzperylene P | 22 | 12 | 5 | 6 | 8 | 45 | 6 |
| 1,2-Benzopyrene | 21 | 10 | 3 | 6 | — | — | — |
| Coronene | 5 | 4 | 3 | 3 | — | — | — |
| Ash (mg.) | 5·8 | 5·1 | 17·0 | 20·1 | — | 19·0 | 7·7 |
| Arsenic P | 11·3 | 6·4 | 15·8 | 8·1 | — | 52·0 | 15·5 |
| Beryllium P | 0·11 | 0·04 | 0·13 | 0·08 | — | 0·59 | 0·23 |
| Molybdenum P | 1·58 | 0·66 | 0·76 | 0·57 | — | 2·81 | 0·52 |
| Chromium | 2·7 | — | 5·6 | 10·5 | — | 5·6 | 1·2 |
| Nickel | 9·0 | 7·5 | 23 | 47 | — | 45 | 3·1 |
| Vanadium | 24·2 | 18·2 | 64 | 254 | — | 28·6 | 2·9 |
| Zinc | 160 | 240 | 196 | 1360 | — | 279 | 107 |
| Total " P " constituents | 65·0 | 31·1 | 25·7 | 21·7 | — | 148·4 | 31·2 |
| Number of stations | 5 | 5 | 5 | 5 | 4 | 4 | 4 |
| Years | 1961–2 | 1961–2 | 1962–3 | 1962–3 | 1954–5 | 1956–8 | 1957 |

Table VI shows very low concentrations of the hydrocarbons in Oslo during the summer with winter/summer ratios far in excess of those in Belfast, Dublin and Helsinki. The ratio of coronene to 3,4-benzopyrene which tends to be low in coal smoke and higher in petrol smoke was greater in summer than in winter in all four cities.

When air pollution is considered in conjunction with the smoking indices Liverpool and Belfast have high rates for both, whichever sex is considered. Dublin has moderate air pollution with high smoking by both sexes ; North Wales has moderate air pollution with moderate smoking by men and low smoking by women ; Copenhagen and Oslo have low air pollution and low smoking rates. The two factors are positively correlated in this series which comprises all the areas for which the necessary data are available, and this makes it rather difficult to distinguish the effects of the two factors upon lung cancer mortality.

TABLE VI.—*Air Pollution by Smoke and Polycyclic Hydrocarbons in Summer and Winter*

| City | Year | Smoke μg./m³ | Hydrocarbons (μg./1000 m³) 3,4-Benzopyrene | 1,12-Benzperylene | 1,2-Benzopyrene | Coronene | Ratio of Coronene to 3,4-Benzopyrene |
|---|---|---|---|---|---|---|---|
| | | | | Summer (April–September) | | | |
| Belfast | 1962 | 79 | 9 | 10 | 10 | 2 | 0·22 |
| Dublin | 1962 | 38 | 3 | 4 | 3 | 2 | 0·67 |
| Helsinki | 1963 | 42 | 1·6 | 2·2 | 1·9 | 1·7 | 1·1 |
| Oslo | 1963 | 36 | 0·5 | 0·8 | 0·8 | 0·7 | 1·4 |
| | | | | Winter (October–March) | | | |
| Belfast | 1961/2 | 166 | 51 | 34 | 33 | 7 | 0·14 |
| Dublin | 1961/2 | 111 | 23 | 20 | 18 | 6 | 0·26 |
| Helsinki | 1962/3 | 53 | 5 | 7 | 5 | 4 | 0·8 |
| Oslo | 1962/3 | 103 | 14 | 11 | 11 | 5 | 0·4 |
| | | | | Ratio of Winter to Summer | | | |
| Belfast | | 2·1 | 5·7 | 3·4 | 3·3 | 3·5 | — |
| Dublin | | 2·9 | 7·7 | 5·0 | 6·0 | 3·0 | — |
| Helsinki | | 1·3 | 3·1 | 3·2 | 2·6 | 2·4 | — |
| Oslo | | 2·9 | 28·0 | 13·8 | 13·8 | 7·1 | — |

Table VII shows the correlation coefficients between various pollution indices in Table V and the death rates in Table I. For total smoke the coefficient is 0·79 with the female death rate at ages 35–44, and coefficients around 0·6 are

TABLE VII.—*Correlation Coefficients at Different Ages between Death Rates from Lung Cancer and Indices of Cigarette Smoking and of Air Pollution*

| Factor correlated with death rate at same age group | | Males: No. of areas | Males: Correlation at ages 35– | 45– | 55–64 | Females: No. of areas | Females: Correlation at ages 35– | 45– | 55–64 |
|---|---|---|---|---|---|---|---|---|---|
| Total smoke in air | | 7 | 0·55 | 0·64 | 0·60 | 7 | 0·79 | 0·58 | 0·60 |
| 3,4-Benzopyrene | P | 7 | 0·68 | 0·67 | 0·60 | 7 | 0·85 | 0·67 | 0·51 |
| 1,12-Benzperylene | P | 7 | 0·54 | 0·63 | 0·67 | 7 | 0·84 | 0·65 | 0·47 |
| Arsenic in air | P | 6 | 0·47 | — | 0·65 | 6 | 0·69 | — | 0·57 |
| Beryllium | P | 6 | 0·38 | — | 0·55 | 6 | 0·66 | — | 0·53 |
| Molybdenum | P | 6 | 0·61 | — | 0·69 | 6 | 0·77 | — | 0·58 |
| Chromium | | 5 | — | — | −0·33 | 5 | — | — | −0·03 |
| Nickel | | 6 | — | — | −0·04 | 6 | — | — | −0·12 |
| Total " P " constituents | | 6* | 0·58 | 0·70† | 0·69 | 6* | 0·92 | 0·53 | 0·65 |
| Number of cigarettes smoked on average daily | | 6‡ | 0·80 | — | — | — | — | — | — |
| | | 8 | — | 0·56 | 0·27 | 5 | 0·85 | 0·98 | 0·71 |
| Percentage smoking 20 or more cigarettes daily | | 6‡ | 0·76 | — | — | — | — | — | — |
| | | 8 | — | 0·69 | 0·36 | — | — | — | — |
| | | 6* | — | 0·81 | 0·57 | — | — | — | — |
| Ditto combined with total total " P " in air Weighted 1 : 1 | | 6* | — | 0·80 | 0·82 | — | — | — | — |
| 2 : 1 | | 6* | — | 0·90 | 0·83 | — | — | — | — |
| 3 : 1 | | 6* | — | 0·89 | 0·86 | — | — | — | — |

* The 6 areas are those in Table V for which the combined air pollution index was available.
† When the percentage smoking 20 or more cigarettes daily is held constant this becomes 0·58.
‡ Without Helsinki and Oslo for which no data were obtained at 35–44. If cigarillos are included for Copenhagen the coefficients become 0·97 with average cigarettes per day and 0·95 with percentage smoking 20 or more daily.

found with male rates at each age and with female rates after 45. For 3,4-benzopyrene the correlation with female mortality falls from 0·85 at 35–44 to 0·51 at 55–64, and for 1,12-benzperylene it falls similarly from 0·84 to 0·47, but with male rates the coefficients average 0·65 for the former and 0·62 for the latter and do not change appreciably with age.

Three of the trace elements, arsenic, beryllium and molybdenum show correlations with mortality at 55–64 with levels between 0·5 and 0·7 in both sexes, but nickel and chromium show no relation with the death rates. Zinc and vanadium are not shown owing to the very abnormal levels in Oslo already referred to. These findings agree in general with results from 23 localities in northern England and Wales in which beryllium and molybdenum seemed to be the elements of most consequence in relation with lung cancer, with arsenic, zinc and vanadium showing weak associations (Stocks, 1960).

The two hydrocarbons and three elements denoted by " P " in Table V had combined concentrations as shown in the 6 areas, and Table VII indicates that correlation between this measure of air pollution and male mortality amounted to 0·58 at ages 35–44, 0·70 at 45–54 and 0·69 at 55–64. For females the coefficient at 35–44 is 0·92 and at higher ages about 0·6. For men, however, cigarette smoking seems to be the more important factor before age 55 whereas after that air pollution seems to take precedence as might be expected from the tendency for heavy smoking to affect mortality most at the early ages. Assuming that the proportion of heavy smokers and the " P " index of air pollution are additive in their influence on the male death rate the two factors from Tables III and V have first been expressed in terms of their mean values in the 6 areas and then combined with weights of 1 : 1, 2 : 1 and 3 : 1, the resulting figures being correlated with the death rates yielding the coefficients at the foot of Table VII.

When the associations of cigarette smoking frequency and amounts of the five air pollutants with death rates from lung cancer are thus combined in proportions of 2 or 3 to 1 the correlations for men aged 35–44 exceed those for either factor taken alone and approach the high level of 0·9. At ages 55–64 the coefficient with smoking was 0·57 and with air pollution 0·69 but in combination the correlation increased to about 0·85. Thus the correlation between the male death rate from lung cancer and the total amount of 3,4-benzopyrene, 1,12-benzperylene, arsenic, beryllium and molybdenum in the air was statistically significant by itself at ages 55–64, and when combined with the frequency of heavy smokers with relative weights of 1 to 2 or 3 the correlation became very strong both at those ages and at 45–54.

These findings may be considered to furnish inadequate evidence for a causal effect of carcinogens in the air upon lung cancer because the number of areas investigated was so small. When values obtained in 1953–54 for the combined concentrations of the " P " constituents in 17 towns in Lancashire (excluding Liverpool which has been used in the present study), Cheshire and West Yorkshire are extracted from Tables II and VII of the paper published in 1960 (Stocks, 1960) and correlated with the standardised death rates of males from lung cancer in 1950–53, as given in Table III of that paper, the result is a coefficient of 0·785 which is highly significant. Further evidence for a causal effect of air pollution by coal smoke in 19 countries is obtained in Section 2 below, and indications that the urban excess of lung cancer in English conurbations arises from that cause are found in Section 3.

### (2) RELATION BETWEEN LUNG CANCER DEATH RATES OF MALES IN 19 COUNTRIES AND CONSUMPTION OF CIGARETTES, SOLID AND LIQUID FUELS

The United Nations Organisation has produced annual statistics since 1951 of the consumption of solid and liquid fuels in different countries, measured in metric tons of coal equivalent and in kilograms per head of population, and the data for 1951–52 and 1955–58 have been used in the present study (U.N.O., 1957, 1960). Details of the annual consumption of cigarettes per adult in various countries derived from official trade statistics since 1920, or as far back as they were available, have been assembled by the Tobacco Research Council (Todd, 1963). Death rates from cancer of the lung and bronchus by sex and age have been correlated with those factors for 19 countries where the rates were sufficiently reliable and for which the national data of cigarettes and fuels were available. Calculation of the death rates was facilitated by the compilations of Segi and Kurihara (1962).

Table VIII shows the mean annual death rates per million in 1958–59 for men aged 25–, 35–, 45–, 55–, 65–74, adjusted to correct for differences in age distribution of the population within each 10-year group since the mortality gradient rises steeply. The final columns give ratios between the rate at 55–64 and those at 35–44 and 25–44. The countries have been ranked in order of the cigarette consumption per adult in the year 1952.

TABLE VIII.—*Lung Cancer Death Rates of Men in 19 Countries in 1958–59*

| | Mean annual rates per million† | | | | | Ratio of rates | |
|---|---|---|---|---|---|---|---|
| Country* | 25–34 | 35–44 | 45–54 | 55–64 | 65–74 | 55–64 / 35–44 | 55–64 / 25–44 |
| U.S.A. | 13·31 | 100·5 | 472·6 | 1337 | 2005 | 13·3 | 24·2 |
| Ireland (Eire) | 8·55 | 111·4 | 402·3 | 1130 | 1351 | 10·1 | 19·5 |
| United Kingdom | 22·39 | 183·3 | 897·4 | 2822 | 3998 | 15·4 | 28·3 |
| Finland | 10·58 | 110·5 | 767·2 | 2647 | 4061 | 24·0 | 45·2 |
| Canada | 5·11 | 49·7 | 344·1 | 1071 | 1640 | 21·5 | 34·0 |
| Switzerland | 8·26 | 77·2 | 475·0 | 1304 | 1923 | 16·9 | 31·5 |
| Netherlands | 14·10 | 96·2 | 593·8 | 1847 | 2364 | 19·2 | 34·5 |
| Australia | 8·67 | 60·0 | 305·4 | 1100 | 1771 | 18·3 | 33·1 |
| New Zealand | 12·58 | 49·8 | 385·7 | 1175 | 2117 | 23·6 | 38·6 |
| Japan | 4·48 | 21·3 | 97·5 | 349 | 695 | 16·4 | 27·5 |
| Austria | 17·34 | 58·8 | 571·8 | 2217 | 3343 | 37·7 | 59·0 |
| South Africa | 12·09 | 77·1 | 390·6 | 1373 | 2062 | 17·8 | 31·7 |
| Denmark | 1·98 | 74·0 | 399·2 | 1264 | 1664 | 17·1 | 37·3 |
| Belgium | 12·91 | 79·2 | 569·8 | 1624 | 2015 | 20·5 | 36·3 |
| Italy | 12·20 | 64·4 | 377·4 | 1026 | 924 | 15·9 | 27·6 |
| France | 8·16 | 53·9 | 317·5 | 969 | 1258 | 18·0 | 31·8 |
| Sweden | 7·06 | 36·8 | 153·4 | 636 | 918 | 17·3 | 29·8 |
| Germany, West | 10·13 | 63·1 | 448·1 | 1623 | 2159 | 25·7 | 45·6 |
| Norway | — | 23·1 | 157·9 | 659 | 578 | 28·5 | 61·2 |

* Ranked in order of cigarette consumption per adult in 1952.
† Corrected for age distribution of population within each group.

Table IX gives the annual cigarette consumption per adult (over 15) in years 1937, 1942, 1947, 1952 and 1957, and the mean annual consumption of solid fuel in 1951–52 and 1955–58 measured in kg. *per capita*, and of liquid fuels in 1955–58 measured on a scale of coal equivalents of the various fuels. The constituent countries of the United Kingdom have been combined since separate data were not available. Consumption of cigarettes per adult in 1952 ranged from 3490 in the U.S.A. to 340 in Norway, and in most countries except the U.S.A., Ireland, Finland, South Africa, Denmark and Norway the rate was still rising in 1957. Consumption of solid fuel per person in 1951–52 ranged from 4212 kg. in the United Kingdom to 239 in Italy, and in most countries the rate had fallen 5 years later, the only notable exceptions being South Africa and West Germany. For liquid fuel the rate was highest in the U.S.A., Canada, Sweden, Denmark, Norway, Australia and New Zealand.

TABLE IX.—*Consumption of Cigarettes and of Solid and Liquid Fuels in* 19 *Countries at Various Dates*

| Country* | Annual cigarette consumption per adult 1939 | 1942 | 1947 | 1952 | 1957 | Solid fuel—mean annual kg. per head 1951–52 | 1955–5 | Liquid fuel kg. per head 1955–5 |
|---|---|---|---|---|---|---|---|---|
| U.S.A. | 1710 | 2330 | 3150 | 3490 | 3440 | 3041 | 2260 | 3168 |
| Ireland (Eire) | 1300 | 1260 | 2070 | 2660 | 2430 | 740 | 621 | 506 |
| United Kingdom | 1790 | 2340 | 2270 | 2320 | 2590 | 4212 | 4129 | 743 |
| Finland | 1480 | 1340 | 1290 | 1850 | 1850 | 611 | 625 | 522 |
| Canada | 860 | 1230 | 1700 | 1780 | 2720 | 2157 | 1708 | 2590 |
| Switzerland | (820) | 820 | 1430 | 1730 | 1920 | 614 | 580 | 716 |
| Netherlands | 710 | (710) | 780 | 1700 | 1750 | 1638 | 1589 | 852 |
| Australia | 520 | 650 | 780 | 1500 | 2040 | 2530 | 2396 | 1138 |
| New Zealand | 690 | 830 | 1570 | 1490 | 1860 | 712 | 638 | 996 |
| Japan | 910 | 1160 | 380 | 1430 | 1630 | 545 | 565 | 205 |
| Austria | 790 | 1620 | 580 | 1280 | 1520 | 1116 | 1231 | 405 |
| South Africa | 630 | 940 | 1100 | 1240 | 1220 | 1825 | 2226 | 258 |
| Denmark | 540 | 590 | 770 | 1210 | 1220 | 1488 | 1290 | 1188 |
| Belgium | 810 | 510 | 1280 | 1180 | 1410 | 3542 | 2748 | 780 |
| Italy | 580 | 650 | 660 | 1180 | 1480 | 239 | 232 | 383 |
| France | 560 | 360 | 760 | 1040 | 1290 | 1667 | 1713 | 575 |
| Sweden | 370 | 440 | 690 | 950 | 1050 | 1095 | 742 | 1967 |
| Germany, West | 750 | (500) | 280 | 720 | 1250 | 2610 | 3047 | 318 |
| Norway | 380 | 260 | 650 | 540 | 550 | 534 | 411 | 1093 |

* Ranked in order of consumption of cigarettes per adult in 1952.
Numbers in parentheses are estimates from adjacent years.

Table X and Fig. 3 show the correlations between the lung cancer death rates of men at different ages in 1958–59 and the cigarette consumption at intervals before that. In 1937, 21 years before, the average cigarette level was 853 and by 1957 it was twice as great. The coefficients with smoking indices 6 and 11 years before were around 0·3 at ages under 35 and over 55, and about 0·4 at 45–54, but these could result from the high correspondence in ranking of the countries by smoking prevalence at the various dates from 1937 to 1952. Thus the smoking levels in 1937 were correlated to the extent of about 0·75 with those in 1947 and 1952 so that partial coefficients between death rates at most ages in 1958–59 and cigarette indices in 1947 and 1952 were slightly negative when the 1937 levels were held constant. This is not true however of the coefficients at ages 35–44 in 1958–59 which were 0·68 and 0·67 with smoking levels in 1952 and 1947, too large to be explained in such a way. When the death rates are related to amounts of smoking prevalent 16 and 21 years before, the coefficients are large and average 0·75 at 35–44, 0·61 at 45–54, 0·53 at 55–64 and 0·61 at 65–74. These figures suggest that the time interval between cigarette smoking and its effect on the

**Fig. 3.**—Lung cancer death rates of men in various countries related to consumption of cigarettes and solid fuel by their populations.

(a) Death rate with cigarette consumption 17, 12, 7 years before, holding solid fuel consumption constant.

(b) Death rate with solid fuel consumption, holding cigarettes 17, 12, 7 years before constant.

17 years 12 years 7 years

(c) Death rates at 35–44, 55–64 in 1958–9 and cigarette consumption in 1947.

death rate from lung cancer was most commonly about 15–20 years but that among men who died at an early age it could be less than this.

In the lower part of the table the changes in cigarette consumption in the 19 countries during successive periods of 5 years have been related to the changes in death rates at 25–34 and 35–44 which occurred 5, 10, 15 or 20 years later between 1952–53 and 1957–58 and between 1957–58 and 1962–63. Considering first the changes in smoking prevalence from 1937 to 1942, these were not reflected to an appreciable extent in the mortality gradients over 5 years which occurred 15–20 years later. For the next period 1942 to 1947 when cigarette consumption was rising more rapidly in most countries, the mortality changes registered 10–15 years later show positive correspondence with the movements in smoking levels, and this is evident also for the young men who were aged 15–24 in the period 1947 to 1952. Apart from the latter group, however, changes in smoking levels after 1947 were not followed by corresponding changes in mortality 5–10 years

FIG. 4.—Hypothetical proportions susceptible to lung cancer amongst men born at various dates and rates of dying from the disease.

(a) Cigarettes per adult consumed annually in United Kingdom.
(b) Surviving susceptibles (after $L$ years) in cohorts of 100,000 born 1896–, 1901–, 1906–, 1911– according to hypothesis.
(c) Lung cancer deaths of males at different ages per annum as proportion of surviving susceptibles in the cohorts.
(d) Total lung cancer deaths of males since birth as proportion of those who have susceptible.

TABLE X.—*Correlation Coefficients between Lung Cancer Death Rates of Men in 1958–59 and Previous Cigarette Consumption per Unit of Population over 15 Years of Age, in Various Years, in 19 Countries*

| Cigarettes: Year | Cigarettes: Average number | Mean interval to 1958–9 | Correlation with death rate in 1958–59 at: 25–34 | 35–44 | 45–54 | 55–64 | 65–74 |
|---|---|---|---|---|---|---|---|
| 1937 | 853 | 21 | — | 0·770 | 0·663 | 0·508 | 0·612 |
| 1942 | 976 | 16 | — | 0·734 | 0·575 | 0·547 | 0·612 |
| 1947 | 1167 | 11 | 0·323 | 0·669 | 0·395 | 0·279 | 0·309 |
| 1952 | 1542 | 6 | 0·288 | 0·682 | 0·408 | 0·285 | 0·328 |

| Period of change | Amount of increase (mean) | Mean interval before death | Ages at time of smoking change | Correlation with rise in death rate during two 5-year periods: 1952–3 to 1957–8: 25–34 | 1952–3 to 1957–8: 35–44 | 1957–8 to 1962–3: 25–34 | 1957–8 to 1962–3: 35–44 |
|---|---|---|---|---|---|---|---|
| 1937 | 77 | 20 | 5–14 | | | −0·100 | |
| to | | 20 | 15–24 | | | | −0·135 |
| 1942 | | 15 | 10–19 | +0·020 | | | |
| | | 15 | 20–29 | | +0·086 | | |
| 1942 | 191 | 15 | 10–19 | | | +0·160 | |
| to | | 15 | 20–29 | | | | +0·388 |
| 1947 | | 10 | 15–24 | +0·275 | | | |
| | | 10 | 25–34 | | +0·155 | | |
| 1947 | 375 | 10 | 15–24 | | | +0·273 | |
| to | | 10 | 25–34 | | | | −0·162 |
| 1952 | | 5 | 20–29 | −0·286 | | | |
| | | 5 | 30–39 | | +0·033 | | |
| 1952 | 207 | 5 | 20–29 | | | −0·546 | |
| to | | 5 | 30–39 | | | | −0·340 |
| 1957 | | | | | | | |

later, the coefficients being negative. This analysis again suggests that men who were under 35 years of age when cigarette consumption was rising rapidly were so affected by the increase as to exhibit corresponding increases in their lung cancer rates after intervals of 10–15 years. This selective effect on early deaths is seen also in the ratios of mortality at 55–64 to those at 35–44 given in the last columns of Table VIII and shown in Fig. 3. When correlated with cigarette consumption in the various countries the ratios in 1958–59 give a negative coefficient of 0·467 with the smoking indices in 1947, and a similar negative coefficient of 0·490 with the indices in 1952. This agrees with the finding in Section 1.

Table XI and Fig. 3 show the correlations between consumption *per capita* of solid and liquid fuels in 1955–58 (and of solid fuel also in 1951–52) as given in Table IX with the death rates from lung cancer in 1958–59 in the 19 countries. There was no correspondence between solid fuel in 1951–52 and cigarette consumption at that time, but solid fuel in 1955–58 was related positively with cigarette consumption in 1942, 1947 and 1952 to the extent of 0·390, 0·320 and 0·131, and correction for this has been made by partial correlation so as to separate the associations with air pollution from those with smoking. The relations between death rates and consumption of liquid fuel are zero or slightly negative. With solid fuel consumption 7 years previously the coefficients, after correction for differing cigarette consumption at the time, were 0·68 at 35–44, falling to 0·47, 0·45 and 0·41 at the next three age groups. With solid fuel consumption during the 5 years preceding 1958–59 correction for cigarette levels in 1942 and 1947 reduced the coefficients somewhat but there remained significant relations of the order of 0·4 at ages over 45 and of 0·5 or greater at ages 35–44.

TABLE XI.—*Correlation Coefficients between Lung Cancer Death Rates of Men in 1958–59 and Consumption per Head of Solid and Liquid Fuels in Various Years in 19 Countries, with Corrections for Cigarette Consumption Levels*

| Factors | Years | Mean annual kg. per head | Correlation with death rate at 35–44 | 45–54 | 55–64 | 65–74 |
|---|---|---|---|---|---|---|
| Solid fuel | 1951–52 | 1627 | 0·527 | 0·450 | 0·443 | 0·402 |
| | 1955–58 | 1513 | 0·578 | 0·485 | 0·487 | 0·474 |
| Liquid fuel | 1955–58 | 968 | −0·055 | −0·138 | −0·199 | −0·143 |
| | | Factor held constant | Partial oefficient with death rate | | | |
| Solid fuel | 1951–52 | Cigarettes 1952 | 0·681 | 0·472 | 0·450 | 0·411 |
| | 1955–58 | ,, 1942 | 0·467 | 0·346 | 0·355 | 0·322 |
| | 1955–58 | ,, 1947 | 0·504 | 0·412 | 0·488 | 0·416 |
| | 1955–58 | ,, 1952 | 0·675 | 0·477 | 0·474 | 0·460 |
| Cigarettes | 1942 | Solid 1955–58 | 0·677 | 0·479 | 0·443 | 0·526 |
| | 1947 | fuel 1955–58 | 0·625 | 0·289 | 0·141 | 0·189 |
| | 1952 | ,, 1955–58 | 0·743 | 0·397 | 0·255 | 0·302 |
| | | Relative weights | Correlation between combination of factors and death rates | | | |
| Cigarettes in 1952 and solid fuel in 1955–58 | | 2 : 1 | 0·840 | 0·590 | 0·493 | 0·507 |
| | | 1 : 1 | 0·808 | 0·603 | 0·533 | 0·530 |
| | | 1 : 2 | 0·752 | 0·576 | 0·531 | 0·515 |
| Cigarettes in 1942 and solid fuel in 1955–58 | | 2 : 1 | 0·760 | 0·633 | 0·624 | 0·655 |
| | | 1 : 1 | 0·782 | 0·643 | 0·621 | 0·634 |

Conversely, correction for the solid fuel consumption produced partial coefficients between death rates in 1958–59 and cigarette consumption in 1942 of 0·68 at ages 35–44 and around 0·5 at later ages. It appears that both factors were related to the death rates independently and to a similar extent, and at the foot of the table the result of combining the two factors with different weights is shown. The multiple effect of combining smoking levels in 1952 with solid fuel levels in the years preceding death in a ratio of 2 : 1 was to produce coefficients of 0·76, 0·63, 0·62 and 0·65 at the four ages, and if equal weighting was used the result was almost the same. At ages 35–44 even higher multiple coefficients resulted when smoking levels in 1942 (that is 16 years before the death period) were used, ranging from 0·84 with weights of 2 : 1 to 0·75 when the ratio was 1 : 2.

### (3) URBAN EXCESS OF MORTALITY FROM CANCERS OF THE LUNG AND STOMACH AND FROM BRONCHITIS IN ENGLISH CONURBATIONS SINCE 1921

The existence of a pronounced urban excess in death rates from the three diseases in England and Wales has been commented upon by epidemiologists for many years but the reasons for the cancer differences have remained in some doubt. Better diagnosis and earlier rise in cigarette smoking frequency in the large towns could account for part of the urban excess in lung cancer up to about 1945, but these factors must have so diminished in importance since then as to now account for only a small part of the excess. Stomach cancer and bronchitis have always shown strong gradients of mortality when the population was divided into social classes on an occupational and economic basis. Recent studies have left no doubt also that bronchitis mortality is much affected by coal smoke as well as by cigarette smoking, whilst stomach cancer though apparently affected to some extent by the former is not related with the latter. Study of the behaviour of urban/rural ratios for these diseases by sex and age since 1921 in several regions of England should help to indicate to what extent the urban excess in lung cancer rates, which persists, is attributable to air pollution by coal smoke.

Cancer deaths by sex and age in county boroughs and the surrounding counties during 1921–30, 1931–39 and 1940–46 had been extracted by the General Register Office for the purposes of various studies of the geographical distribution of cancers of the lung, stomach and other sites (Stocks, 1936, 1939, 1950, 1952, 1958) ; and the bronchitis deaths for those years have been compiled from the Registrar General's Statistical Reviews. From 1950 onwards the division into conurbations and remainders has made the urban ratios not quite comparable with those in 1921–46 since the conurbations incorporate some urban districts and because county boroughs not in the conurbation had to be included in the remainder of the region. The effect of these differences was to increase the urban excess for lung cancer somewhat whilst stomach cancer ratios were hardly affected, as may be seen from a comparison of the two measures of " urban " in the Northern Region for years 1940–46.

Table XII shows the urban ratios in the Northern Region of England in 1921–46 obtained by expressing the death rates in all county boroughs in terms of those for the rest of the region taken as 100, and in 1950–62 by relating the death rates in Tyneside conurbation as a whole to those for the region excluding the conurbation. At ages where the numbers of deaths were too small in 1921–30 ratios are not shown, and where data for ages 45–54 and 55–64 could not be separated the groups are combined. At the foot of the table the arithmetical differences between the ratios for lung and stomach cancer are given, the stomach ratios providing a measure of the effects of social class selection between town and country which affects all three causes of death.

Any urban effect of more cigarette smoking there may have been before 1940 would have affected bronchitis as well as lung cancer, and in 1931–39 the urban ratios for these diseases were similar for males though higher for bronchitis in females. In 1950–62 the ratios for both causes were around 140 for each sex after 45, and since any urban smoking excess would no longer account for more than a 10 per cent difference (Buck and Brown, 1964) the high ratios can only be attributed to air pollution together with social class differences. Comparison between the lung and stomach cancer ratios in 1950–57 shows however that social selection also could have accounted for only a small part of the ratio differences, and at ages over 45 the high urban ratios for lung cancer are inexplicable apart from air pollution.

Table XIII gives the urban ratios during 1921–46 in the West Riding of Yorkshire, obtained by expressing the death rates in the county boroughs of the West Yorkshire conurbation in terms of those in the rest of the county taken as 100, and similar ratios are shown for the period 1954–63 which relate to the whole conurbation. In 1931–39 the urban excess was more pronounced for lung cancer than for bronchitis and this has been true also in recent years. The differences

TABLE XII.—*Urban Ratios for Mortality from Cancer of the Lung and Stomach and Bronchitis in the Northern Region of England since* 1921

| Cause of death | Period | Males 25– | 45– | 55– | 65– | 75+ | Females 25– | 45– | 55– | 65– | 75+ |
|---|---|---|---|---|---|---|---|---|---|---|---|
| | | (a) All county boroughs % of remainder of region | | | | | | | | | |
| Lung cancer | 1921–30 | .. | 131 | 163 | 96 | 90 | .. | .. | .. | .. | .. |
| | 1931–39 | 156 | 145 | 142 | 120 | 118 | 149 | 122 | 117 | 83 | 63 |
| | 1940–46 | 136 | 167 | 172 | 154 | 136 | 193 | 176 | 185 | 170 | 138 |
| Bronchitis | 1921–30 | 192 | 160 | | 135 | 126 | 130 | 173 | | 143 | 130 |
| | 1931–39 | 137 | 169 | 135 | 142 | 126 | 125 | 143 | 172 | 144 | 121 |
| Stomach cancer | 1921–30 | .. | 120 | 109 | 102 | 75 | .. | 114 | 107 | 96 | 98 |
| | 1931–39 | 130 | 135 | 104 | 116 | 103 | 91 | 126 | 120 | 99 | 90 |
| | 1940–46 | 127 | 165 | 114 | 112 | 100 | 117 | 120 | 118 | 111 | 108 |
| | | (b) Conurbation county boroughs % of remainder of region | | | | | | | | | |
| Lung cancer | 1940–46 | 154 | 172 | 173 | 177 | 166 | 128 | 170 | 199 | 186 | 288 |
| Stomach cancer | 1940–46 | 126 | 142 | 120 | 115 | 91 | 109 | 109 | 122 | 107 | 118 |
| | | (c) Conurbation (all districts) % of remainder of region | | | | | | | | | |
| Lung cancer | 1950–57 | 127 | 142 | | 156 | 148 | 97 | 148 | | 142 | 122 |
| | 1958–62 | 131 | 145 | | 153 | 144 | 77 | 137 | | 142 | 167 |
| Bronchitis | 1950–57 | 114 | 158 | | 150 | 142 | 137 | 117 | | 146 | 134 |
| | 1958–63 | 85 | 132 | | 129 | 156 | 254 | 163 | | 147 | 137 |
| Stomach cancer | 1950–57 | 127 | 123 | | 98 | 100 | 99 | 113 | | 110 | 110 |
| | 1958–62 | 117 | 118 | | 109 | 99 | 103 | 97 | | 96 | 97 |
| | | Difference between lung and stomach cancer ratios | | | | | | | | | |
| Urban ratio | 1921–30 | .. | +11 | +54 | − 6 | +12 | .. | .. | .. | .. | .. |
| for lung cancer | 1931–39 | +26 | +10 | +38 | + 4 | +15 | +58 | − 4 | − 3 | −16 | −25 |
| compared with | 1940–46(a) | + 9 | + 2 | +58 | +42 | +36 | +76 | +56 | +67 | +59 | +30 |
| ratio for | 1940–46(b) | +28 | +30 | +53 | +62 | +75 | +19 | +61 | +77 | +79 | +170 |
| stomach cancer | 1950–57 | 0 | +19 | | +58 | +48 | − 2 | +35 | | +32 | +12 |
| | 1958–62 | +14 | +27 | | +44 | +45 | −26 | +40 | | +46 | +70 |

between the ratios for lung and stomach cancers up to 1951 were much larger than any local variations in cigarette smoking could account for.

Comparison between the two large conurbations in the North West Region and the remainder of that region during 1954–63 shows that the lung cancer ratios average 143 whereas those for stomach cancer average 106, and this difference like those in the West Riding would seem to be explicable only by the higher levels of air pollution in the cities comprising the conurbations of South-east Lancashire and Merseyside. When Greater London death rates at ages over 45 are related to those in the rest of the London and South Eastern Region taken as 100, the result is as follows during the period 1950–57 :

| | Males 45– | 65– | 75+ | Females 45– | 65– | 75+ |
|---|---|---|---|---|---|---|
| Lung cancer | 122 | 144 | 158 | 132 | 140 | 170 |
| Bronchitis | 172 | 196 | 190 | 164 | 225 | 183 |
| Stomach cancer | 119 | 116 | 115 | 132 | 128 | 118 |

Here the urban ratios for lung cancer average 145, for bronchitis 188 and for stomach cancer 121, the difference between lung and stomach not being as pro-

TABLE XIII.—*Urban Ratios for Mortality from Cancer of the Lung and Stomach and from Bronchitis in Yorkshire (West Riding) since* 1921, *and in the North West Region of England in* 1954–63

| Cause of death | Period | Males 25– | 45– | 55– | 65– | 75+ | Females 25– | 45– | 55– | 65– | 75+ |
|---|---|---|---|---|---|---|---|---|---|---|---|
| | | West Yorkshire conurbation county boroughs % of remainder of the county | | | | | | | | | |
| Lung cancer | 1921–30 | 175 | 187 | 180 | 177 | | .. | 126 | 175 | 99 | |
| | 1931–39 | 149 | 190 | 163 | 156 | | .. | 186 | 182 | 148 | |
| | 1940–46 | 191 | 168 | 161 | 175 | | .. | 115 | 133 | 164 | |
| | 1950–51 | .. | 157 | | 163 | | | 127 | | 101 | |
| Bronchitis | 1921–30 | 201 | 163 | | 117 | 115 | 162 | 155 | | 123 | 115 |
| | 1931–39 | 147 | 165 | 154 | 102 | 85 | 89 | 105 | 94 | 98 | 96 |
| Stomach cancer | 1921–30 | 122 | 128 | 105 | 109 | 106 | 118 | 101 | 96 | 93 | 87 |
| | 1931–39 | 101 | 100 | 99 | 100 | 98 | 107 | 93 | 91 | 84 | 79 |
| | 1940–46 | 120 | 108 | 115 | 123 | | 93 | 108 | 100 | 100 | |
| | 1947–51 | .. | 110 | | 100 | | .. | 102 | | 94 | |
| | | West Yorkshire conurbation (all districts) % of remainder of West Riding | | | | | | | | | |
| Lung cancer | 1954–63 | 133 | 126 | | 131 | 127 | 126 | 116 | | 134 | 102 |
| Bronchitis | 1954–63 | 121 | 116 | | 117 | 117 | 105 | 107 | | 119 | 106 |
| | | Difference between lung and stomach cancer ratios | | | | | | | | | |
| Lung cancer ratio compared with stomach cancer ratio | 1921–30 | +53 | +59 | +75 | +67 | | .. | +25 | +77 | + 7 | |
| | 1931–39 | +48 | +90 | +64 | +57 | | .. | +93 | +91 | +66 | |
| | 1940–46 | +71 | +60 | +46 | +52 | | .. | + 7 | +33 | +64 | |
| | 1950–51 | .. | +47 | | +63 | | .. | +25 | | + 7 | |
| | | North West Region conurbations % of remainder of the region | | | | | | | | | |
| Lung cancer | 1954–63 | 139 | 140 | 132 | 134 | 156 | 182 | 121 | 118 | 142 | 167 |
| Stomach cancer | 1954–63 | 100 | 123 | 113 | 105 | 111 | 108 | 99 | 97 | 102 | 98 |
| Difference | | +39 | +17 | +19 | +29 | +45 | +74 | +22 | +21 | +40 | +69 |

nounced as in the northern regions as might be expected from the more extensive area of the conurbation and the nature of its industries and air pollution.

### (4) EVIDENCE THAT ONLY THOSE WHO HAVE FIRST DEVELOPED A SUSCEPTIBILITY TO CANCER OF THE LUNG ARE AFFECTED BY SUCH FACTORS AS CIGARETTE SMOKING AND AIR POLLUTION

In the original plan for the survey of smoking and air pollution described in Section 1 it was hoped to carry it out in 20 or more cities. An objection advanced after the start was that air pollution levels in many towns have been changing in recent years and their relative values as now measured may not be reliable indications of the relative pollution levels during the years when lung cancer was being initiated among the city residents who are now dying of the disease. The validity of this objection stems from the belief that an interval of twenty years or more elapses between the initiation of the cancer by the action of some substance in tobacco smoke or city air and the resulting death from bronchogenic cancer, but this is an unproven hypothesis which has been shaken by some recent studies. The prevailing view has been stated thus by Clemmesen (1965) : " Most authors accept the assumption of an average latent period of at least 20 years between the beginning of smoking and the development of bronchial carcinoma ". Supposing this to be a valid assumption, it does not follow however that the amount of exposure to smoke during the period 15–20 years before death had been more important in determining when the final stages of the disease would be reached than the amount of exposure during a period only a few years before death occurred. Two studies have shown for instance that the risk of dying from lung cancer is much smaller among men who have stopped smoking cigarettes than it is in comparable groups who have continued to smoke, and this reduction of risk becomes evident soon after the cessation of smoking.

Thus Hammond (1962) found among smokers of 20 or more cigarettes per day that 8588 men who had stopped smoking for 5–9 years suffered a death rate of 72, and 10,788 who had stopped for 10 years or more had a rate of 12, compared with 137 among those who had continued to smoke. Doll and Hill (1964) found a similar reduction of risk among doctors who had stopped smoking. These findings seem to be incompatible with a supposition that smoking starts a process of cancer development which then after a latent period of some 20 years results in the appearance of clinical symptoms regardless of exogenous factors operating during that period. There are many differences between this and another hypothesis, to which little attention has been paid, that cigarette smoking and inhalation of certain substances in air accelerate a process which has been started in the lung already by some endogenous agency. If smoking acts as an accelerator in those persons who are in the latent stage of lung cancer the effects could appear in a few years among heavy smokers who had been in the latent stage for a sufficiently long time. Cessation of the exogenous irritant might then slow down the process of growth, postponing appearance of clinical signs and death from the disease to a later age or allowing death to intervene from some other cause before the cancer had become clinically apparent.

According to the latter hypothesis a limited proportion of the population at any moment have been for short or long periods in the latent stage of lung cancer, initiated by an unknown agency, and of these "susceptibles" some expose themselves to smoking or air pollution which hasten progression of the disease to death at an earlier age than may result among the susceptibles not so exposed, many of whom would never reach the clinical stage owing to prolongation of the latent condition. One result of this would be that the proportion of deaths which occur at the younger ages would tend to be greater in cities or countries where heavy cigarette smoking is more prevalent than in cities or countries with low smoking indices, and this is found to be the case in the studies described in Sections 1 and 2.

In Belfast and Dublin with high rates of cigarette smoking about 20 per cent of lung cancer deaths of men occur between ages 25 and 55 compared with 12 per cent in Oslo where the smoking rates are low, though the expected proportions based on age distributions of the populations are approximately the same in each city. This appeared also from the remarkable correspondence between the relative slope of the mortality graph between ages 45 and 65 and the proportion of men aged 45–54 who had smoked 20 or more cigarettes daily, and by the fact that the correlation between smoking indices and the death rate in men falls as age advances. In the 19 countries the ratios of death rates at 55–64 to those at 35–44 were negatively correlated with the cigarettes being consumed per adult in those countries about 10 years previously.

If only limited numbers of people are becoming susceptible to lung cancer it might happen in a city with high levels of smoking and also of air pollution that the number of heavy smokers at risk would become so depleted by their high mortality as to cause a levelling out of the normal upward trend of death rates with increasing amount of smoking. Such an effect was observed in Liverpool (Stocks, 1958) and the following conclusion was stated on p. 124 : " The regression lines of mortality on maximum weekly cigarettes ever smoked habitually run parallel up to about 200 weekly for the different geographical areas, but the urban graph for Liverpool then ceases to rise further, suggesting that despite great exposure to irritant factors not all men are susceptible to lung cancer ". This was the first pointer to the hypothesis now to be formulated and discussed.

Another fact favouring the hypothesis is the large excess of lung cancer in men compared with women which is found universally. Although this has been attributed to the fact that women began to smoke cigarettes in quantity at later dates than did men, and the sex difference has been used as an argument against air pollution being an important factor, there are difficulties in accepting such reasons. For example, it has been shown (Stocks, 1958) that in Liverpool the standardised death rates at ages 35–74 in women were less than one third of those in men who recorded the same smoking frequency, whether they had been smoking 100 cigarettes or more per week, fewer than 100 per week or none at all, and this suggests that a smaller proportion of women than of men is susceptible to cancer of the lung. There is also the obvious objection to the current theory of causation that out of the large numbers of men who have smoked heavily for over 20 years only a small proportion develop lung cancer. In Dublin for example at least a third of the men aged 55–64 in the population record heavy cigarette smoking for 30 years or more but only about 4 per cent of all men of that age had a statistical likelihood of dying from lung cancer.

*Mathematical basis for the hypothesis*

In 1953 an analysis of death rates from stomach cancer in England and Wales which occurred in " cohorts " of men and women who had been born in successive periods of time and who died between 1921 and 1950 produced a curve depicting the mean rate of dying from that cause at each age from birth onwards (Stocks, 1953). This was compared with curves resulting from a simple mathematical formula based on a supposition that $c$ successive cell changes, happening with an annual probability $q$, were necessary to initiate this form of cancer, after which there would be a latent period whilst the cancer was developing. It was found that if $c$ had the value 5, implying that number of successive cell changes, and if $q$ had the value 0·033 for men, or 0·027 for women, these being the mean probabilities that such a change would occur in a year, good correspondence resulted with the cohort curves for the two sexes when an age interval of about 17 years was allowed between the curves. This interval represented the average latent period between initiation of the cancer and death from it. A theory postulating 5 cell changes as necessary to initiate cancer in general was arrived at independently by Nordling in the same year (Nordling, 1953). It is reasonable to envisage a similar process operating to initiate lung cancer.

In deriving the formula to calculate the rates of initiation at different ages it is assumed for simplicity that not more than one of the successive cell changes would occur in a single year, so cancer initiation would not start until the end of the $c^{th}$ year of life when the proportion of the population becoming affected is given by the $(c + 1)^{th}$ term of the binomial expansion of $(p + q)^c$ where $p = 1 - q$. At the end of the $(c + x)^{th}$ year the proportion becoming affected will be

$$\frac{(c + x - 1)\,(c + x - 2)\ldots c}{1.2.\ldots x}\,p^x\,q^c$$

At the ends of successive years from the $c^{th}$ onwards the proportions are

$$q^c\,;\;\; q^c pc\,;\;\; q^c p^2\,(c + 1)\,c/1.2\,;\;\; q^c p^3\,(c + 2)\,(c + 1)\,c/1.2.3 \text{ etc.}$$

and the multiplying factor when passing from age $a$ to age $a + 1$ is then always $(1 - q)a/(a + 1 - c)$, since $p = 1 - q$, and from this the curve of initiation rates with advancing age can be drawn. Moreover, since there is no reason to suppose any appreciable selection in the risk of dying from causes other than the cancer, the survivors at any age out of a million born will contain the same proportions of persons becoming newly affected as if there had been no mortality, and the resulting curve represents the annual rates of initiation per million living at each age.

Comparison of such curves with rates of dying from lung cancer in cohorts of males born in 5-year periods since 1896–1900 revealed that no correspondence was possible unless $c$ had the value 5 and that the best agreement resulted when $q$ had the value 0·043, in which case there was an interval averaging about 20 years between age at initiation and age at death, but rather shorter when the age at death was under 35 or over 55. The hypothesis supposes that during the latent interval lung cancer is slowly developing to become clinically manifest during the last few years or months of the interval, and that only those who have become susceptible by completion of the initiation process can develop the disease.

Table XIV shows the death rates attributed to lung cancer per million living at various ages among men who had been born at different dates from 1896 to 1920 in England and Wales. The rates were increasing at ages up to 45 in successive cohorts from (a) to (d) but then became steady ; and at 45–54 the increase had ceased by cohort (c) and at 55–64 by cohort (b). The mortality curves coalesced from about 1943 onwards, and the curve of initiation rates obtained from the formula corresponds with the trend of death rates $L$ years later, the average interval $L$ for all males being as shown in the last column of the table.

It is a feasible hypothesis therefore that from the 5th year of life susceptibility to lung cancer arises in some of the males after completion of a series of 5 cell changes occurring with an annual probability 0·043, and that susceptibles are thus added at successive ages to the population according to the initiation rates per million living shown in the table. There would follow a latent period of growth to the stage when it becomes clinically recognisable and finally results in death, the interval $L$ from initiation to death being made up of both latent period and clinical stage. Cigarette smoking or air pollution may act upon the lung or bronchus once the latent stage has advanced sufficiently and may accelerate the growth from that time onwards, resulting in a shorter period than the average and earlier death than would have occurred otherwise. In non-smokers the period $L$ might be much above the average. Such a hypothesis could account for a number of facts which seem hardly compatible with the current theory and it is worth while to examine its implications in more detail.

TABLE XIV.—*Death Rates in England and Wales from Cancer of the Lung and Bronchus amongst Men Born in Years* 1896 *to* 1920, *Compared with Hypothetical Rates of Cancer Initiation*

| Age at death | Death rate per million living in the age group of men born during years: 1896–00 (a) | 1901–05 (b) | 1906–10 (c) | 1911–15 (d) | 1916–20 (e) | Initiation rate of new susceptibles per million at age: 20 years before | *L* years before | Average interval to death for all males *L* |
|---|---|---|---|---|---|---|---|---|
| 15–19 | 2 | 2 | 2 | 2 | 2 | — | — | — |
| 20–24 | 3 | 3 | 4 | 5 | 6 | — | — | — |
| 25–29 | 5 | 7 | 10 | 14 | 16 | 2 | 36 | 15 |
| 30–34 | 11 | 20 | 30 | 34 | 36 | 36 | 85 | 18 |
| 35–39 | 54 | 68 | 81 | 94 | 98 | 158 | 135 | 21 |
| 40–44 | 149 | 191 | 236 | 248 | 249 | 416 | 305 | 21 |
| 45–49 | 384 | 544 | 579 | 580 | 580 | 836 | 650 | 21 |
| 50–54 | 954 | 1244 | 1249 | 1208 | — | 1412 | 1300 | 20 |
| 55–59 | 2003 | 2319 | 2295 | — | — | 2102 | 2280 | 18 |
| 60–64 | 3319 | 3671 | — | — | — | 2927 | 3420 | 17 |
| 65–69 | 4933 | — | — | — | — | 3787 | 4470 | 16 |

TABLE XV.—*Deaths from Lung Cancer and Hypothetical Numbers of Susceptibles in Populations of Successive Cohorts of Males*

| Age group | Mid-populations of cohorts of 10,000 men born at dates stated: 1896–00 (a) | 1901–05 (b) | 1906–10 (c) | 1911–15 (d) | Numbers of deaths in the 5 year period resulting from rates in Table XIV (D)†: (a) | (b) | (c) | (d) |
|---|---|---|---|---|---|---|---|---|
| 15–19 | 7062 | 7304 | 7701 | 7812 | 7 | 7 | 8 | 8 |
| 20–24 | 6941 | 7194 | 7588 | 7701 | 10 | 11 | 15 | 19 |
| 25–29 | 6709 | 7073 | 7453 | 7587 | 17 | 25 | 37 | 53 |
| 30–34 | 6578 | 6951 | 7343 | 7490 | 36 | 70 | 110 | 127 |
| 35–39 | 6437 | 6823 | 7216 | 7402 | 174 | 268 | 292 | 348 |
| 40–44 | 6266 | 6664 | 7072 | 7299 | 467 | 636 | 835 | 905 |
| 45–49 | 6051 | 6471 | 6906 | 7129 | 1162 | 1760 | 2288 | 2067 |
| 50–54 | 5772 | 6209 | 6576 | 6887 | 2753 | 3810 | 4067 | 4157 |
| 55–59 | 5381 | 5800 | 6100 | — | 5389 | 6725 | 7000 | — |
| 60–64 | 4816 | 5177 | — | — | 7992 | 9868 | — | — |
| 65–69 | 4024 | — | — | — | 9925 | — | — | — |

| Age group | Cumulative deaths in cohort up to end of the 5 year period (CD): (a) | (b) | (c) | (d) | Cumulative susceptibles appearing up to *L* years before end of the period* (CS): (a) | (b) | (c) | (d) |
|---|---|---|---|---|---|---|---|---|
| 25–29 | 34 | 46 | 64 | 84 | 124 | 132 | 138 | 142 |
| 30–34 | 70 | 116 | 174 | 211 | 328 | 338 | 366 | 372 |
| 35–39 | 244 | 384 | 466 | 558 | 531 | 563 | 582 | 602 |
| 40–44 | 711 | 1020 | 1301 | 1463 | 1772 | 1881 | 2004 | 2047 |
| 45–49 | 1873 | 2780 | 3589 | 3531 | 3792 | 4268 | 4322 | 4651 |
| 50–54 | 4626 | 6590 | 7656 | 7688 | 9882 | 9409 | 1,0004 | 1,0485 |
| 55–59 | 1,0015 | 1,3315 | 1,4656 | — | 1,8313 | 18536 | 1,9500 | — |

| Age group | Ratio of annual deaths (D/5) to mean susceptibles surviving in the age period: (a) | (b) | (c) | (d) | Ratio of cumulative deaths at end of age period to cumulative susceptibles *L* years before (CD/CS): (a) | (b) | (c) | (d) |
|---|---|---|---|---|---|---|---|---|
| 25–29 | 0·045 | 0·068 | 0·111 | 0·184 | 0·274 | 0·348 | 0·464 | 0·592 |
| 30–34 | 0·041 | 0·091 | 0·165 | 0·232 | 0·213 | 0·343 | 0·475 | 0·567 |
| 35–39 | 0·128 | 0·267 | 0·379 | 0·679 | 0·460 | 0·682 | 0·801 | 0·927 |
| 40–44 | 0·150 | 0·245 | 0·408 | 0·577 | 0·401 | 0·541 | 0·649 | 0·705 |
| 45–49 | 0·156 | 0·299 | 0·637 | 0·487 | 0·491 | 0·649 | 0·830 | 0·759 |
| 50–54 | 0·153 | 0·354 | 0·513 | 0·425 | 0·468 | 0·700 | 0·765 | 0·733 |
| 55–59 | 0·172 | 0·385 | 0·441 | — | 0·547 | 0·718 | 0·752 | — |

† i.e. Rates per million living as in table applied to 100 times the mid-populations of cohort survivors out of 10,000 born.

* Corrected for depletion by general death rate during the *L* interval.

Table XV gives the populations of the successive cohorts at the middle of each age group, calculated on life table principles, the numbers of deaths ($D$) in each age period produced by the death rates in Table XIV when applied to those populations, and the cumulative deaths which would have occurred from lung cancer by the end of the age period. The cumulative susceptibles ($CS$) are obtained by applying the initiation rates at ages $L$ years before each age period of dying to the cohort population of the latter age period (thus allowing for depletion by the general death rate during the latent interval through causes other than lung cancer), and then summing the resulting figures up to the end of the age period. The number of susceptibles surviving at the middle of the age group is taken as the mean of the values of $CS$—$CD$ at the beginning and end of the age interval, and the annual deaths ($D/5$) per 100 surviving susceptibles are shown at the foot of the table. The figures show, for example, that according to the hypothesis before age 55 about ten out of every 100 men born about the beginning of the century would have become liable to die of lung cancer by undergoing the necessary cell changes, and of these 8 would have already died of it.

In the years up to about 1945 two factors were affecting the death rates attributed to lung cancer, increasing completeness of diagnosis of the condition and increasing frequency of cigarette smoking, but from that time onwards those influences had become no longer important. The annual consumption of cigarettes per adult over age 15 in the United Kingdom was 1030 in 1921–25, rising in the following quinquennial periods to 1252, 1456, 1870, 2376 and then ceasing to rise (2266 in 1946–50 and 2384 in 1951–55), until 1956–60 when it increased to 2634, as shown by the graph in Fig. 4. Whilst smoking frequency was rising the effects of each increase were first evident among young persons and then extended to older men, and this is reflected in the ratios of deaths to surviving susceptibles above the broken line in the table which marks the position in 1950. In the cohort (a) of men born in 1896–1900 they were aged 30–50 when smoking frequency was rising rapidly and their fatality ratio of number dying in a year to number at risk remained around 0·15 ; in the next cohort (b) the ratio increased whilst the men were about age 35 and then remained around 0·25 from that age to 50. In cohort (c) born in 1906–10 the ratio increased progressively to 0·41 by age 45 and 0·64 by age 50 ; and in the last cohort (d) born in 1911–15 the ratio rose to 0·68 during 1946–50 when the men were aged 35–39.

After 1950, by which time diagnostic recognition of the disease and smoking frequency among men had become virtually stable, the fatality ratios of annual deaths to surviving susceptibles in the cohort born in 1906–10 fell with advancing age from 0·64 at 45–49 to 0·44 at 55–59, and in those born in 1911–15 it fell from 0·58 at 40–44 to 0·42 at 50–54. This means that the annual rates of dying among the susceptibles at ages 40–, 45–, 50–, 55–59 averaged 58, 56, 47 and 44 per 100 living whereas the death rates at those ages were 25, 58, 123 and 230 per million. According to current ideas the steep rise in mortality rates between age 40 and 60 would be attributed vaguely to an increasing liability of men to develop lung cancer as they grow older, but according to the hypothesis under consideration it has nothing to do with " ageing " but results from the accumulating susceptibles in the population. The numbers of these are rising rapidly with advancing age so that despite an almost constant fatality ratio the total deaths which occur among them also increases.

The hypothesis supposes that the fatality ratios are enhanced by smoking and air pollution which accelerate the fatal termination of the latent stage. In places therefore where there are more heavy smokers this would lead to more deaths at an early age, depleting the numbers of susceptibles surviving to later ages and leaving fewer to die in those age groups. This would lower the ratio of death rate at 55–64 to that at 35–44 as actually observed in Sections 1 and 2 of this paper.

The ratios of cumulative deaths to cumulative number of men in each cohort who had become susceptible to lung cancer $L$ years before the end of each age period are shown at the foot of Table XV, the position about year 1950 being indicated by a broken line. Before that date this ratio tended to rise with advancing age in each cohort but after 1950 it was remarkably constant around 0·75 in the last 3 cohorts. It is reasonable to expect that about a quarter of the men who had become susceptible to lung cancer would by avoidance of the extraneous accelerating factors escape dying of the disease.

*Summary of evidence supporting the hypothesis*

Some of the facts which seem incompatible with current ideas about the role of smoking and air pollution on lung cancer causation but which could be explained by the hypothesis of limited susceptibility and aggravation by extraneous irritants in the late stages of evolution of the cancer are recapitulated below :

(1) In cities such as Dublin where about a third of the men aged 55–64 have smoked over 20 cigarettes per day for 30 years only one tenth of such men die eventually from lung cancer ;

(2) In areas with more heavy smokers and in countries where the level of cigarette consumption had been high 10 years before, the death rates from the disease at ages 35–44 tend to be enhanced relatively to those at 55–64 ;

(3) Whilst the death rate of male cigarette smokers in country areas rises progressively with the intensity of smoking, in one large city with much air pollution the rate ceased to rise when the smoking average exceeded about 30 cigarettes per day ;

(4) The proportion who have smoked 30 or more cigarettes daily among all men dying of lung cancer does not increase with age but tends to fall although the duration of their smoking increases with age ;

(5) The risk of dying from the disease falls off within a few years after cessation of cigarette smoking ;

(6) According to the hypothesis that only a proportion develop a susceptibility the rate of dying among the surviving susceptibles since 1950 has stabilised at about one half annually at ages after 40 and the steep mortality gradient in the whole population as age advances is not attributable to ageing but is explained simply by the increasing number of susceptibles appearing in the population ;

(7) Since 1950 the ratio of cumulative deaths to cumulative susceptibles has, according to the hypothesis, become constant at about three-quarters, implying that one out of four of the men at risk had escaped dying of lung cancer ;

(8) Multiple correlations between death rates from lung cancer and combinations of the consumption of cigarettes and of solid fuel in different countries exceed 0·8 at ages 35–44 among men but are smaller at higher age groups.

In the present climate of opinion the risk at any age of developing lung cancer is supposed to be unalterable for those who have been smoking cigarettes heavily for 20 years, but according to the new hypothesis the risk will be reduced if cigarette smoking is stopped by age 30, and the safest course for youths if they *must* smoke cigarettes would be to smoke not more than 10 per day until age 25 and then change to pipe smoking only, which carries little risk. Current ideas which account in part for the poor response to advice to curb cigarette smoking may be wrong, and if they are they ought to be proved to be so by epidemiological studies designed to settle the matter. Possible rewards in cancer prevention from such studies are large and they ought not to be discouraged by apathy or by the cost and labour they involve.

### SUMMARY

(1) Simultaneous surveys of cigarette smoking and of the amounts of polycyclic hydrocarbons and trace elements in the air throughout a year have been made in 6 European cities and 2 areas of Wales. The results when correlated with the death rates from cancer of the lung and bronchus yielded substantial and independent relations with the smoking and air pollution indices.

(2) When published data from 19 countries of the consumption of cigarettes per adult and consumption of solid and liquid fuels *per capita* in various years were compared with the lung cancer death rates notable correlations were found both with smoking and solid fuel but none with liquid fuel.

(3) Analysis of the death rates from lung and stomach cancer and bronchitis since 1921 in conurbations of England compared with the surrounding regions shows that after allowing for differences in social and other factors a large urban excess of lung cancer remains which must be attributed to air pollution.

(4) When lung cancer death rates in cohorts of men born since 1896 are matched with the numbers expected from a hypothesis that the cancer is started after a series of 5 cell changes which progressively add susceptible individuals to the population according to a probability formula, good agreement results if there is an average latent interval between initiation and death of 21 years in mid-life or rather less before 35 and after 55. If smoking and air pollution act by accelerating the final stages of growth in those susceptibles who have reached an advanced point in the latent interval, this would explain many observed facts which are not compatible with the current view that everyone is liable to lung cancer.

The author wishes to thank the Dublin Health Authority, Professor J. Pemberton (Belfast), Dr. R. Pedersen (Oslo), Professor E. Saxén (Helsinki), the Medical Research Council's Air Pollution Unit, Warren Spring Laboratory (Department of Scientific and Industrial Research), the General Register Office, the British Empire Cancer Campaign for Research and the Health Officers of the areas surveyed for their help and co-operation in these studies.

### REFERENCES

BUCK, S. F. AND BROWN, D. A.—(1964) *Tobacco Research Council, London*, Research paper No. 7.

CAMPBELL, J. M. AND CLEMMESEN, J.—(1956) *Dan. med. Bull.*, November, p. 205.

CLEMMESEN, J.—(1965) 'Statistical Studies in the Aetiology of Malignant Neoplasms'. Copenhagen (Munksgaard).

DOLL, R. AND HILL, A. B.—(1964) *Br. med. J.*, i, 1397.

HAMMOND, E. C.—(1962) *Bull. Inst. int. Statist.*, **39**, 437.
LINDHARDT, MARIE—(1960) 'The Sickness Survey of Denmark 1951–54'. Copenhagen (Munksgaard).
NORDLING, C. O.—(1953) *Br. J. Cancer*, **7**, 68.
SEGI, M. AND KURIHARA, M.—(1962) 'Cancer Mortality for Selected Sites in 24 Countries', No. 2. 1958–59. Tohoku University, Japan.
STOCKS, P.—(1936) *Rep. Br. Emp. Cancer Campn*, **13**, 240.—(1939) *Rep. Br. Emp. Cancer Campn*, **16**, 308.—(1950) *Br. J. Cancer*, **4**, 147.—(1952) *Br. J. Cancer*, **6**, 99.—(1953) *Br. J. Cancer*, **7**, 407.—(1958) *Supplement to 35th Rep. Br. Emp. Cancer Campn*—(1960) *Br. J. Cancer*, **14**, 397.
STOCKS, P., COMMINS, B. T. AND AUBREY, K. V.—(1961) *Int. J. Air Wat. Pollut.*, **4**, 141.
TODD, C. F.—(1963) *Tobacco Research Council, London.* Research paper No. 6.
U.N.O.—(1957) 'World Energy Supplies'. U.N. Statistical Papers, Series J, No. 2.—(1960) 'World Energy Supplies'. U.N. Statistical Papers, Series J, No. 3.